# Nanotechnology in inflammation: cutting-edge advances in diagnostics, therapeutics and theranostics

**DOI:** 10.7150/thno.91394

**Published:** 2024-04-08

**Authors:** Yuting Liu, Ziqi Lin, Yuting Wang, Liuhui Chen, Yuequan Wang, Cong Luo

**Affiliations:** 1 Department of Pharmaceutics, Wuya College of Innovation, Shenyang Pharmaceutical University, Shenyang 110016, P. R. China.; 2 Joint International Research Laboratory of Intelligent Drug Delivery Systems, Ministry of Education, Shenyang Pharmaceutical University, Shenyang 110016, P.R. China.

**Keywords:** inflammation, biomedical nanotechnology, precise diagnosis, anti-inflammatory nanotherapeutics, nanotheranostics

## Abstract

Inflammatory dysregulation is intimately associated with the occurrence and progression of many life-threatening diseases. Accurate detection and timely therapeutic intervention on inflammatory dysregulation are crucial for the effective therapy of inflammation-associated diseases. However, the clinical outcomes of inflammation-involved disorders are still unsatisfactory. Therefore, there is an urgent need to develop innovative anti-inflammatory strategies by integrating emerging technological innovations with traditional therapeutics. Biomedical nanotechnology is one of the promising fields that can potentially transform the diagnosis and treatment of inflammation. In this review, we outline recent advances in biomedical nanotechnology for the diagnosis and treatment of inflammation, with special attention paid to nanosensors and nanoprobes for precise diagnosis of inflammation-related diseases, emerging anti-inflammatory nanotherapeutics, as well as nanotheranostics and combined anti-inflammatory applications. Moreover, the prospects and challenges for clinical translation of nanoprobes and anti-inflammatory nanomedicines are highlighted.

## Introduction

Inflammation is generally considered as a defense mechanism to protect the body from external stimuli and invasions 1. However, long-term inflammatory reactions can lead to the dysfunction of cells, tissues, organs and living systems, and also increase the risks of chronic diseases 2. Therefore, timely identification and expeditious intervention are essential for the effective management of chronic inflammatory disorders 3. Recent advancements in nanoparticle drug delivery systems (nano-DDSs) offer the potential to revolutionize both diagnostic and therapeutic approaches to inflammation intervention 3. Nanosensors and nanoprobes can facilitate the detection, monitoring and imaging of inflammatory lesions 3. Moreover, elaborately engineered nano-DDSs can endow anti-inflammatory nanomedicines with a multitude of benefits, including improving unfavorable physicochemical properties, prolonging the systemic circulation time and reducing off-target drug toxicity 4. In recent decades, considerable endeavors have also been made to achieve the synchronized co-delivery of two or more probes and/or therapeutic agents within a nano-DDS for theranostics and combination therapy of inflammatory diseases 5.

### Inflammation-related diseases

**Figure 1:**
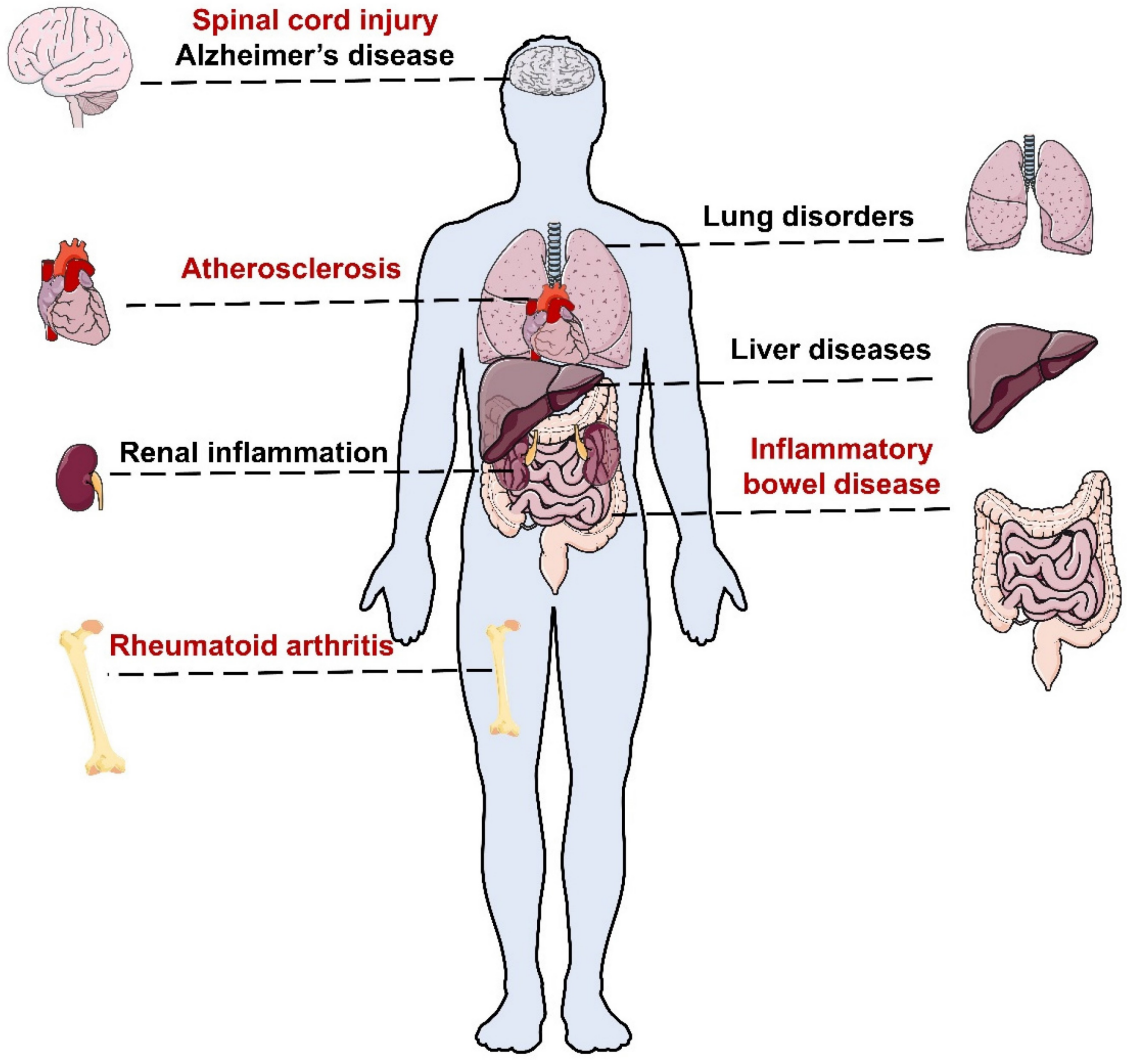
Schematic representation of inflammatory-related diseases in different organs.

Acute inflammation is usually accompanied by redness, swelling, fever, pain, dysfunction and other clinical symptoms with pathological changes, whereas chronic inflammation is often asymptomatic in early stages, posing challenges for early detection 2. Chronic inflammation can affect different organs of the body and cause various types of inflammatory diseases (Figure 1). Without proper treatment, chronic inflammation can result in severe diseases such as atherosclerotic cardiovascular disease (ASCVD) 6, diabetes 7, degenerative diseases 8 and even cancer 9.

Rheumatoid arthritis (RA) is a chronic and complex autoimmune disease that affects about 0.5-1.0% of the population 10. According to the Global Burden of Disease (GBD) study, 17.6 million people suffered from RA worldwide in 2020, and this number is projected to rise to 31.7 million people by 2050 10. RA usually begins gradually with pain and swelling in polyarticular joints 10. Without adequate treatment, RA can cause serious complications including cardiovascular (CV), pulmonary, gastrointestinal, and neurological diseases 11, 12.

Inflammatory bowel disease (IBD) includes Crohn's disease and ulcerative colitis 13. According to the 2019 GBD study, about 4.9 million individuals worldwide had IBD and 35,600 died from it 13. IBD often causes non-specific symptoms in the early stages such as abdominal pain, diarrhea and weight loss. However, long-term inflammation can result in serious and irreversible intestinal damage and increase the risk of colorectal cancer 13.

Atherosclerosis (AS) is a condition in which plaques build up inside the arteries, narrowing them and reducing blood flow to vital organs, which can lead to many heart diseases and strokes 14, 15. Among them, ASCVD is the most common and deadly complication of AS 16. The 2019 Global Burden of Disease (GBD) study estimated that AS affected 226.7 million people and caused 2.9 million deaths worldwide 16.

Lung disorders such as asthma, pneumonia and pulmonary fibrosis (PF) are also major causes of morbidity and mortality worldwide, affecting people of all ages. Asthma is a common chronic inflammatory disease that affects over 300 million people globally 17. Pneumonia is a frequent and potentially fatal complication of Coronavirus Disease 2019 (COVID-19), which can cause fluid accumulation and inflammation in the lungs 18. Notably, COVID-19-associated pneumonia often involves both lungs and may persist even after recovery from the infection 18.

In addition to the above disorders, inflammation can also affect other organs and tissues, such as the brain, liver and kidney. Neuroinflammation is a process of inflammation in the nervous tissue, which is involved in the development of Alzheimer's disease (AD), spinal cord injury (SCI) and other neurological disorders 19. Liver and renal inflammation can also result in severe outcomes, such as liver failure, cirrhosis, kidney failure and chronic kidney disease, without proper treatment 20, 21.

### Cytokines and pathways of inflammation

Inflammation is a complex and dynamic process that involves various inflammatory cytokines 22. Inflammatory cytokines can be classified into two categories: pro-inflammatory and anti-inflammatory. Pro-inflammatory cytokines, such as tumor necrosis factor alpha (TNF-α), interleukins (e.g., IL-1, IL-6, IL-12) and interferons (e.g., IFN-α, IFN-γ), promote inflammation and immune responses, while anti-inflammatory cytokines, such as interleukins (e.g., IL-4, IL-10, IL-13) and transforming growth factor beta (TGF-β), suppress inflammation and regulate immune tolerance 22. The balance between pro-inflammatory and anti-inflammatory cytokines is essential for maintaining a healthy immune system and preventing inflammatory-related diseases 23. Excessive pro-inflammatory cytokines can lead to chronic inflammation and tissue damage. Inflammatory cytokines can also activate different signaling pathways by binding to specific receptors on target cells, as shown below 22.

Oxidative stress results from an imbalance between the generation and removal of reactive oxygen species (ROS) in the body 24. ROS are unstable molecules that can damage cellular structures and functions, such as DNA, membranes and proteins 24. Oxidative stress can induce pro-inflammatory cytokines and contribute to various inflammatory diseases, such as diabetes, asthma and RA, by modulating and amplifying several molecular pathways, including nuclear factor kappa B (NF-κB) and mitogen-activated protein kinases (MAPKs), which regulate the expression of genes involved in inflammation 24.

The NF-κB pathway plays a central role in inflammation, as it induces the transcription of pro-inflammatory genes, such as cytokines, chemokines, immunoreceptors, cell adhesion molecules and regulators of apoptosis 25. Dysregulation of the NF-κB pathway may cause chronic inflammation disorders, such as autoimmune diseases, IBD, RA and cancer 25.

The Janus kinase (JAK)/activator of transcription (STAT) pathway is a key signaling pathway that transmits the effects of various cytokines and growth factors to the nucleus 26. By regulating the expression of genes related to inflammation and immunity, this pathway is involved in various inflammatory diseases, such as RA, IBD and asthma 26.

Toll-like receptor (TLR) signaling is a process that enables the immune system to detect and respond to pathogens and damaged cells 27. TLRs recognize specific molecular patterns in microbes or host cells and activate signaling pathways, leading to the production of inflammatory molecules such as cytokines, chemokines and interferons 27. These molecules recruit and activate immune cells, which initiate inflammatory responses and tissue repair 27. However, excessive or prolonged TLR signaling can also cause tissue damage and chronic inflammation, contributing to conditions such as infections, autoimmune disorders, allergies and cancer 27.

The MAPK pathway is also a key regulator of inflammation, as it governs the production and release of pro-inflammatory cytokines and chemokines, such as TNF-α 28. In addition, the MAPK pathway modulates the activation of transcription factors, such as NF-κB 28. Dysregulation of the MAPK pathway can lead to chronic inflammation and even cancer 28.

### Current diagnosis for inflammation

Accurate diagnosis is an important prerequisite for effective treatment of diseases. Inflammation is a common indicator of many diseases, but it can vary in type and location. Therefore, different methods are needed to diagnose inflammation in different parts of the body. Some of the most common methods are blood tests and imaging tests.

#### Blood tests

Blood tests are the primary method to indicate inflammation, employing various markers that reflect the level and type of inflammatory response. Some of these markers, such as erythrocyte sedimentation rate (ESR) and white blood cell count (WBC), have been used for inflammation detection for over fifty years 29. However, they are not highly specific and may be influenced by other factors 29. In recent years, more sensitive and accurate serum markers have emerged and become widely available in clinical practice. For instance, C-reactive protein (CRP) is the foremost and widely used inflammatory marker, particularly effective at identifying infections, as it can increase up to 1000-fold from its basal plasma/serum levels within a 19-hour half-life 30. Additionally, procalcitonin (PCT) levels in healthy individuals are usually below 0.05 μg/L, but they can surge dramatically within hours during severe inflammation 31. Furthermore, IL-6, as an early-phase marker, is also valuable for monitoring early inflammation 29.

While expedient and straightforward, these tests do not pinpoint the precise cause or site of inflammation 32. Moreover, their reliability can be influenced by other factors, such as medications, infections or chronic diseases 32. Therefore, they are generally not sufficient and need to be interpreted along with imaging modalities.

#### Imaging modalities

Imaging modalities have been developed for years to diagnose inflammation-related diseases in preclinical and clinical studies. For instance, bioluminescence imaging of inflammation is facilitated by the reaction of luminol with myeloperoxidase (MPO) in inflamed regions 33. Additionally, ultrasound (US) can measure plaque inflammation by evaluating enhanced permeability and neovascularization using non-targeted microbubbles. Furthermore, computed tomography (CT) is the most commonly used clinical imaging method among other imaging modalities and injectable iodinated compounds are employed as contrast agents 34. Other imaging methods, such as radionuclide imaging (RI) and magnetic resonance imaging (MRI) usually utilize ^18^F fluorodeoxyglucose (FDG) and Gd^3+^ complex as contrast agents in the clinic, respectively 35, 36.

Despite widespread application, these imaging approaches still have certain shortcomings, including (i) Inferior penetration and sensitivity of bioluminescence imaging 37; (ii) Limited sensitivity of US imaging in soft tissues 38; (iii) Inadequate spatial resolution and safety concerns of CT and RI 39; and (iv) Low detection resolution of MRI in some organs, such as liver, lung and gastrointestinal tract 40.

### Current treatment for inflammation

Inflammatory diseases require not only precise diagnosis but also timely and effective drug therapy. A variety of drugs have been developed for inflammation treatment.

#### Chemical drugs

Non-steroidal anti-inflammatory drugs (NSAIDs) are a group of medicines that can reduce pain, fever and inflammation 41. There are many types of NSAIDs, such as aspirin, ibuprofen, naproxen, diclofenac, celecoxib and etoricoxib 42. They work by blocking the enzymes that produce prostaglandins, which are chemicals that cause inflammation and pain in the body 42. However, NSAIDs have several limitations that affect their clinical efficacy and safety. These include unfavorable physicochemical properties, low *in vivo* delivery efficiency and potential for off-target toxicity, which induce some risks and side effects, such as stomach ulcers, bleeding, high blood pressure, kidney problems and heart problems 42.

Corticosteroids including dexamethasone (Dex) are a class of synthetic drugs that can reduce inflammation and suppress the immune system 43. They are used to treat various conditions, such as asthma, allergies, eczema, RA and autoimmune diseases 43. However, long-term or high-dose use of corticosteroids can cause serious side effects that outweigh their benefits. These include weight gain, mood changes, high blood pressure, diabetes, osteoporosis and an increased risk of infections 43.

#### Gene drugs

Gene therapy is a novel approach to treat or prevent diseases by modifying genes within the body's cells 44. It has potential applications for various inflammatory conditions, such as cystic fibrosis, arthritis and chronic pain 45. However, gene therapy for inflammation faces several hurdles that need to be overcome before it can be widely used in the clinic. These include instability, immunogenicity and off-target toxicity of the gene delivery vectors, which can cause unwanted immune reactions, gene mutations or side effects 46. Therefore, more research and clinical trials are needed to ensure the safety and efficacy of gene therapy for inflammation.

#### Protein and peptide drugs

Some protein drugs, such as infliximab, adalimumab and certolizumab, have been approved for marketing and can be used to treat inflammatory diseases, such as RA and IBD 47-49. Peptide drugs, such as semaglutide (Rybelsus^®^) and octreotide (MYCAPSSA^®^), are also used for inflammation treatment, as they can regulate insulin secretion and treat diabetes 50. However, protein and peptide drugs have some drawbacks, such as instability, easy degradation, immunogenicity, side effects and high cost 51, 52. Therefore, a pressing need for developing effective drug delivery methods to enhance anti-inflammatory therapy.

### Nanotechnology-driven diagnosis and treatment

Nanotechnology has been widely applied in biomedicine, especially for developing nano-DDSs to treat inflammation-related diseases 53-58. The size, surface charge and shape of nanoparticles (NPs) are crucial factors that affect their interactions with biological systems 59. Moreover, surface modification is also an important aspect in the design of NPs to enhance their targeting and circulation 59.

The size of NPs determines their interactions with various tissues and organs in the body 60. Smaller NPs (< 10 nm) can cross the blood-brain barrier more easily than larger ones 60. NPs (< 100 nm) tend to accumulate in the alveoli, while those > 200 nm are cleared by alveolar macrophages 60. NPs (> 100 nm) are also prone to be captured by the reticuloendothelial system in the liver, spleen and lymph nodes 60. Smaller NPs (6-100 nm) have a higher chance of penetrating the blood vessel wall and reaching inflammation sites than larger ones owing to their favorable contribution and permeability 61. NPs (100-200 nm) may have lower efficiency in reaching inflammation sites, but they exhibit longer circulation and better selective retention at inflammatory sites 59. Moreover, NPs < 6 nm may face renal excretion or clearance 60.

The surface charge of NPs has a significant impact on their behavior in biological environments. Cationic NPs are typically internalized more efficiently than neutral or anionic ones 62. However, the high positive charge (> 15 mV) could increase the adsorption of plasma proteins, forming protein coronas that reduce their targeting and biocompatibility 62. On the other hand, negatively charged NPs can accumulate more in serum, prolonging the retention time of the drug carrier due to lower charge-selective filtration 62.

The shape of NPs influences their cellular uptake 59. Typically, spherical NPs have higher fluidity and stability, but are also easily cleared by phagocytic cells 59. Non-spherical NPs, such as rod-shaped, sheet-shaped or star-shaped, have larger surface area and more functional groups, which enhance the drug loading and density of target ligands 59. For instance, the cellular internalization of methylpolyethylene glycol-coated anisotropic gold NPs in RAW264.7 cells showed shape-dependent preferences for various endocytosis pathways 63. The efficiency of cellular uptake increased from stars, to rods, to triangles 63.

Surface modification improves the *in vivo* circulation and targeting of NPs 64. For instance, polyethylene glycolylation (PEGylation), especially with PEG molecular weight over 2000 Da, helps NPs evade clearance by macrophages, leading to longer circulation in the body 64. In addition, some targeted ligands on NPs' surfaces can bind to specific receptors or molecules on the cell surface, resulting in selectively delivery of anti-inflammatory agents 65. For example, mannose acts as a targeted ligand, binding to the mannose receptor expressed on activated macrophages and immune cells 65. In a mouse model of RA, mannose-decorated poly(lactic-co-glycolic) acid (PLGA) NPs loaded with methotrexate (MTX), a potent anti-inflammatory drug, exhibited enhanced accumulation in inflamed joints and reduced arthritis severity compared to free MTX or non-targeted NPs 65.

Compared with conventional formulations, nano-DDSs have the following advantages based on the scale effect of nanostructures, including (i) Improving the pharmacokinetics of drugs by changing their physicochemical properties (e.g., water solubility, lipid solubility) and helping them cross physiological and pathological barriers, thus enhancing their bioavailability 66, 67; (ii) Exploiting the unique physical, chemical, optical and biological properties of nanomaterials for biosensing and imaging, thus providing real-time monitoring and feedback of the disease status and treatment outcome 68; (iii) Endowing nanomedicines with multiple functionalities by modifying the nanocarriers, prolonged circulation and intelligent drug release, thus enhancing the precision and efficiency of the therapy 69; (iv) Co-delivering two or more probes and/or therapeutic agents for combined theranostics of inflammatory diseases, thus achieving synergistic effects and overcoming drug resistance 70.

### Significance of this review

Recent reviews on nanotechnology applications for inflammation have highlighted key areas including diagnostics, therapeutics and theranostics. For instance, Tu *et al.* offer a comprehensive overview of biomaterials for inflammation control 43. However, their review may not encompass all nanobiomaterials and lack detailed examples 43. Similarly, Han *et al.* provide a novel perspective on metal-based NPs for inflammation control 71. Nevertheless, their review only focuses on the specific metal-based NPs for treating inflammatory diseases 71. Moreover, numerous studies have shown promising results in nanotechnology-based diagnosis and treatment of inflammation in recent years 54, 72-74. Consequently, a systematic review is warranted to provide the latest developments in emerging nanotechnology-based approaches for diagnosing and managing chronic inflammation.

**Figure 2:**
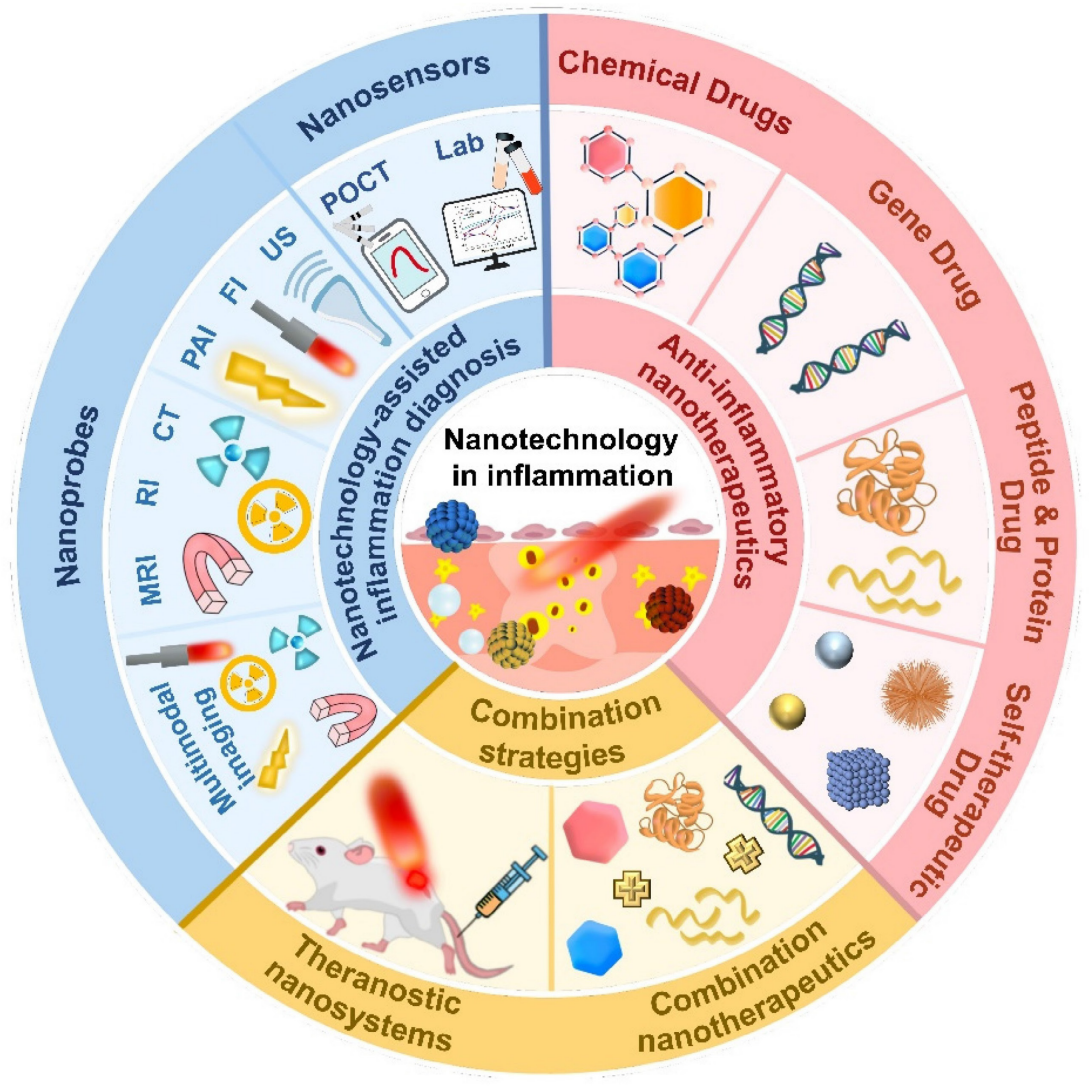
Graphic scheme of advancements in nano-delivery systems for diagnosing and treating inflammation.

In this review, we aim to provide a timely overview of the latest developments in inflammation diagnosis and treatment (Figure 2), highlighting the advances and challenges of nanotechnology-based methods for inflammation diagnosis, therapeutics and theranostics. First, various nanotechnology-based diagnostic methods were pointed out via emphasizing their benefits, applications and prospects in inflammation diagnosis. Then, a large quantity of emerging anti-inflammatory nanotherapeutics were provided to elucidate the design principles of different nanocarriers and compare their advantages and disadvantages in anti-inflammatory therapy. Furthermore, biomedical nanotechnology-driven theranostics and combination therapy of inflammation were also discussed. Eventually, we shed light on the potential and hurdles pertaining to the clinical translatability of anti-inflammatory nanomedicines.

## Nanotechnology-assisted inflammation diagnosis

Chronic inflammation is often asymptomatic in the early stage, but it can lead to serious diseases if left untreated 75. Therefore, early inflammation detection is of importance for accurate confirmation of inflammatory lesions and timely disease intervention 76. Nanotechnology-driven solutions are increasingly being applied in inflammation diagnosis 77. Their high sensitivity and specificity enable the detection of biomarkers associated with inflammation at incredibly low concentrations 78. Additionally, nanotechnologies not only improve imaging accuracy and sensitivity but offer real-time monitoring for assessing inflammation levels in the body 79. Herein, we discuss nanobiosensors and imaging nanoprobes in diagnosing inflammation by comparing their advantages, disadvantages and clinical application prospects.

### Nano-biosensing

Biosensors are analytical devices used to detect and measure biological molecules or substances within a sample. Biosensors typically consist of analytes, bioreceptors, signal transducers and display panels 80. While traditional clinical detection methods such as indirect immunofluorescence (IIF), western blotting and enzyme-linked immunosorbent assay (ELISA) have been crucial in diagnostics, they often face challenges related to standardization and scalability. Nanotechnology-based biosensors have revolutionized diagnostics by leveraging nanomaterials and structures to detect biomarkers with incredible precision and sensitivity (Table 1) 81. These biosensors utilize nanoscale components, such as NPs, nanowires or nanotubes, to detect specific biological molecules or signals, including autoantibodies, genetic markers, inflammatory factors and complements 81, 82.

Additionally, unlike complex, time-consuming hospital or lab tests, point-of-care testing (POCT) nanosensing offers user-friendly interfaces, rapid detection and cost-effective diagnosis, holding great promise in diagnosis of inflammatory-related diseases 83.

#### Nano-biosensors in lab

Recently, nanomaterials such as quantum dots (QDs), carbon dots (CDs) and gold NPs (AuNPs) have revolutionized biosensor technology by amplifing signals and improving biocompatibility due to their chemical, electrical, optical, mechanical or magnetic properties. These nanomaterials can further improve sensitivity and reduce side effects of biosensors, making them more suitable for inflammation diagnosis.

QDs are innovative fluorescent nanomaterials that enable the development of efficient biosensors with high sensitivity, selectivity, rapidity and simplicity 90. Their unique optical and electronic properties, including high brightness, photostability, broad absorption spectrum, tunable emission spectrum and distinctive photoelectrochemical activity, contribute to their effectiveness 90. When integrated into functionalized sensing systems, QDs can successfully detect various inflammation biomarkers, such as CRP 91, PCT 92 and TNF-α 93, showing great potential for diagnosing and monitoring inflammation. For instance, Cai *et al*. constructed a photoelectrochemical biosensor for ultra-sensitive “on-off” detection of inflammation biomarkers: TNF-α and methylase (MTase) 93. The biosensor combines selenide (WSe_2_) nanoflowers, AgInS_2_ (AIS)/ZnS QDs and DNA nanostructures. The (AIS)/ZnS QDs, acting as excellent photosensitive materials, matched the energy level of WSe_2_ nanoflowers, boosting the photocurrent signal by 65 times compared to WSe_2_ nanoflowers alone. Additionally, (AIS)/ZnS QDs serve as signal amplifiers and specific recognition elements for the target molecules. Such a nanobiosensor showed a linear range of 0.1-1000 pg/ml for TNF-α and 0.01-100 U/ml for MTase, with a limit of detection (LOD) of 0.03 pg/ml for TNF-α and 0.003 U/ml for MTase, indicating high efficiency and sensitivity in inflammation detection 93. In another study, Lv* et al*. systematically investigated the influence mechanism of metal ions on QD fluorescence signal amplification and discovered that Ca^2+^ could not only increase the fluorescence intensity of QDs, but facilitate the binding efficiency of antigen-antibody 94. Compared with the common QD-fluorescence-linked immunosorbent assay (QD-FLISA), Ca^2+^-QD-FLISA showed ultra-high detection sensitivity of CRP, an inflammation biomarker, by 4 times, reaching 0.23 ng/ml. The ion-QD-FLISA method was then successfully extended to use other ions, including Mg^2+^, Ba^2+^, Fe^2+^ and Mn^2+^ as well as applied to detect other biomarkers such as serum amyloid A (SAA) and PCT. The amalgamation of metal ions with QDs presents a simple and effective approach for the early detection of inflammation 94.

CDs are versatile fluorescent nanomaterials prized for their adjustable luminescence and excellent biocompatibility in biosensing applications 95. Immunomagnetic CDs are a class of carbon-based nanomaterials that integrate magnetic functionalities and specific antibodies 95. They are valuable in analytical chemistry and biosensing due to their ability to recognize and isolate specific targets efficiently without complex sample preparation procedures 95. For instance, Liu *et al.* employed a fluorescence amplification system using nanocapsules and magnetic CDs to detect PCT 95. They proposed two different sensing strategies: the magnetic separation strategy and the homogeneous immunoassay strategy. The magnetic separation strategy used immunonanocapsules as sensors and immunomagnetic CDs as capture probes, achieving ultra-sensitive trace detection of PCT through normal immune reaction and magnetic separation, with a detection range of 1-1000 pg/ml and an LOD of 0.3 pg/ml. While the homogeneous immunoassay strategy utilized immunonanocapsules as energy transmitters and immunomagnetic CDs as sensors, achieving direct rapid detection of PCT through the principle of fluorescence resonance energy transfer (FRET), with a detection range of 0-100 ng/ml and an LOD of 0.41 ng/ml. In conclusion, the former strategy is suitable for complex samples and the later suitable for rapid detection. Both strategies exhibit stability and reliability in achieving accurate quantification of PCT, with better linear relationship and lower detection limit than the traditional chemiluminescence microparticle immunoassay (CMIA) method 95.

AuNPs are widely used for electrochemical signal amplification in immunosensors due to their large surface area, strong catalytic activity, biocompatibility, and ability to improve electron transfer rates and create an optimal microenvironment for capture antibodies 96. For instance, Yola *et al.* reported a novel voltammetric immunosensor for the detection of TNF-α, using AuNPs as part of the sensor platform (AuNPs/S-MWCNTs) and the signal enhancer (bimetallic Ni/Cu-MOFs) 96. The capture TNF-α antibody was attached to the sensor platform through amino-gold affinity to achieve immunoreaction. The proposed immunosensor showed high sensitivity, selectivity, stability and reproducibility and the detection time was less than 30 minutes without complex sample labeling or multiple washing steps, providing an effective method for the diagnosis and monitoring of TNF-α-related diseases 96. In addition, Wang *et al.* introduced an electrochemiluminescent (ECL) immunosensor for highly sensitive detection of lipoproteinassociated phospholipase A2 (Lp-PLA2), an AS biomarker, via a multifunctional nanoplatform (AuNPs@CoFe PBA) consisting of CoFe prussian blue analogue (PBA) and AuNPs 87. The AuNPs@CoFe PBA's exceptional peroxidase-like activity significantly amplified the ECL signal by approximately 29-fold owing to the synergistic effect between PBA and AuNPs. Additionally, the abundant AuNPs presented more active sites for immobilization of antibody proteins, enhancing the sensor response. Upon capturing Lp-PLA2, the sensor emitted a reduced ECL signal due to increased mass and electron transfer resistance. Under optimized conditions, the developed ECL immunosensor exhibited a wide linear detection range from 1 to 2200 ng/ml and an LOD of 0.21 ng/ml. 87.

#### Nano-biosensors in POCT

**Figure 3:**
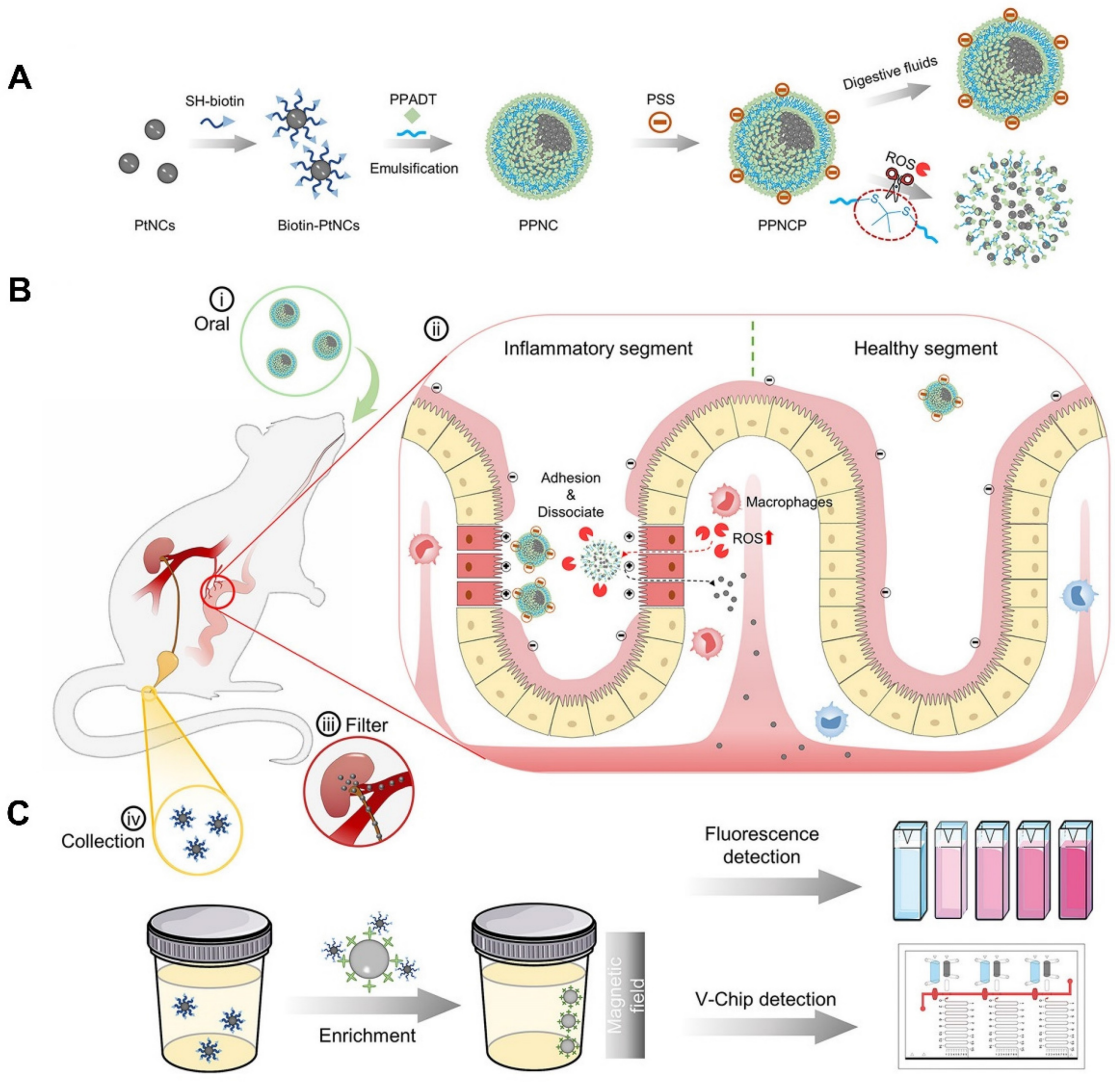
**An oral nanosensor for IBD monitoring. (A)** The preparation of PPNCP nanosensor, which is composed of PtNCs encapsulated by a ROS-responsive polymer. **(B)** The release of ROS-sensitive PPNCP supernanoparticles in inflamed intestine. **(C)** The collection of biotin-PtNCs by magnetic enrichment and the detection of by fluorescence and V-Chip assay. Adapted with permission, from 84 Copyright 2021 American Chemical Society.

POCT is a diagnosis strategy that has been widely used for disease diagnosis and treatment monitoring outside the laboratory in recent years 97. Unlike complex and time-consuming tests in hospitals and laboratories that often require specialized equipment and trained personnel, POCT shows many benefits such as convenient, efficient and cheap, especially for large-scale disease diagnostics 98. The World Health Organization (WHO) has established the criteria for POCT sensing platforms, including sensitivity, specificity, user-friendliness and rapidity (delivering results within 30 minutes) 99. Nanomaterials are suitable for enhancing signal transduction and amplifying sensor response to improve performance of biosensors, thus holding great promise in POCT 99. As shown in Figure 3, Zhou *et al.* constructed a non-invasive nanosensor (PPNC@PSS) that could disassemble into ultrasmall platinum nanoclusters (PtNCs) within inflammatory microenvironments associated with IBD 84. To form supernanoparticles (PPNC), poly (1,4-phenyleneacetone dimethylene thioketal) with ROS-responsiveness was employed to facilitate encapsulation of PtNCs, which was further modified with Poly-(styrenesulfonate) to form PPNC@PSS and targeted inflamed intestinal sites. Upon oral administration, PPNC@PSS responded to elevated ROS levels in inflamed intestines, releasing small PtNCs (2 nm) that were filtered into urine and then detected by simple and sensitive urine readers, such as fluorescence and volumetric bar-chart chips (V-Chip). Notably, nanosensor showed higher accuracy and sensitivity than conventional fCAL-based ELISA assays, due to the amplified signals from both urinary biological enrichment and PtNCs' catalytic activity. Such an adaptable nanosensor holds great promise for home-care applications to personalize the assessment of diseases and follow-up therapeutic efficacy 84.

Recently, a flexible amperometric immunosensor targeting MPO has been developed using a modified electrode with colloidal QDs (CQDs) 85. CQDs have exceptional surface characteristics that enable them to bind directly and stably to protein surfaces, facilitating the conversion of antigen-antibody specific binding reactions into detectable electrical currents 85. The portable nanobiosensor offered precise quantitative analysis of the MPO with high sensitivity (LOD = 31.6 fg/ml) and exhibited impressive stability during short-term storage at low temperatures 85.

Wearable biosensor devices can monitor various physiological parameters, such as blood pressure, glucose level and sweat composition, by attaching to the human body or clothing 100. They offer a potential solution for diagnosis and monitoring due to their convenience, reusability and sensitivity 3. For example, Wang* et al.* presented a flexible and regenerative biosensor using a graphene-Nafion composite membrane to detect cytokine storm biomarkers in undiluted biofluids linked to inflammation 101. Among them, graphene as a two-dimensional (2D) nanomaterial, demonstrated excellent electrical conductivity, mechanical properties and biocompatibility, and could enhance the transmission and amplification of electrical signals. Additionally, Nafion was a fluorinated polymer, showing good ion exchange properties and anti-biofouling ability, and could protect the active molecules on the electrode surface. Then, the graphene-Nafion film was modified with specific aptamers for biomarkers. This nanobiosensor not only exhibited high consistency and sensitivity in detecting biomarkers (IFN-γ) with an LOD 740 fM in undiluted human sweat, but could withstand multiple regenerative cycles (up to 80) by simple washing and regeneration steps and 100 cyclic crumpling tests without mechanical failure. What's more, the detection data could be uploaded in real-time to the cloud or mobile devices via wireless communication technology, enabling remote monitoring and intervention for doctors and patients, thus enhancing medical efficiency and quality 101.

### Nano-imaging

**Figure 4:**
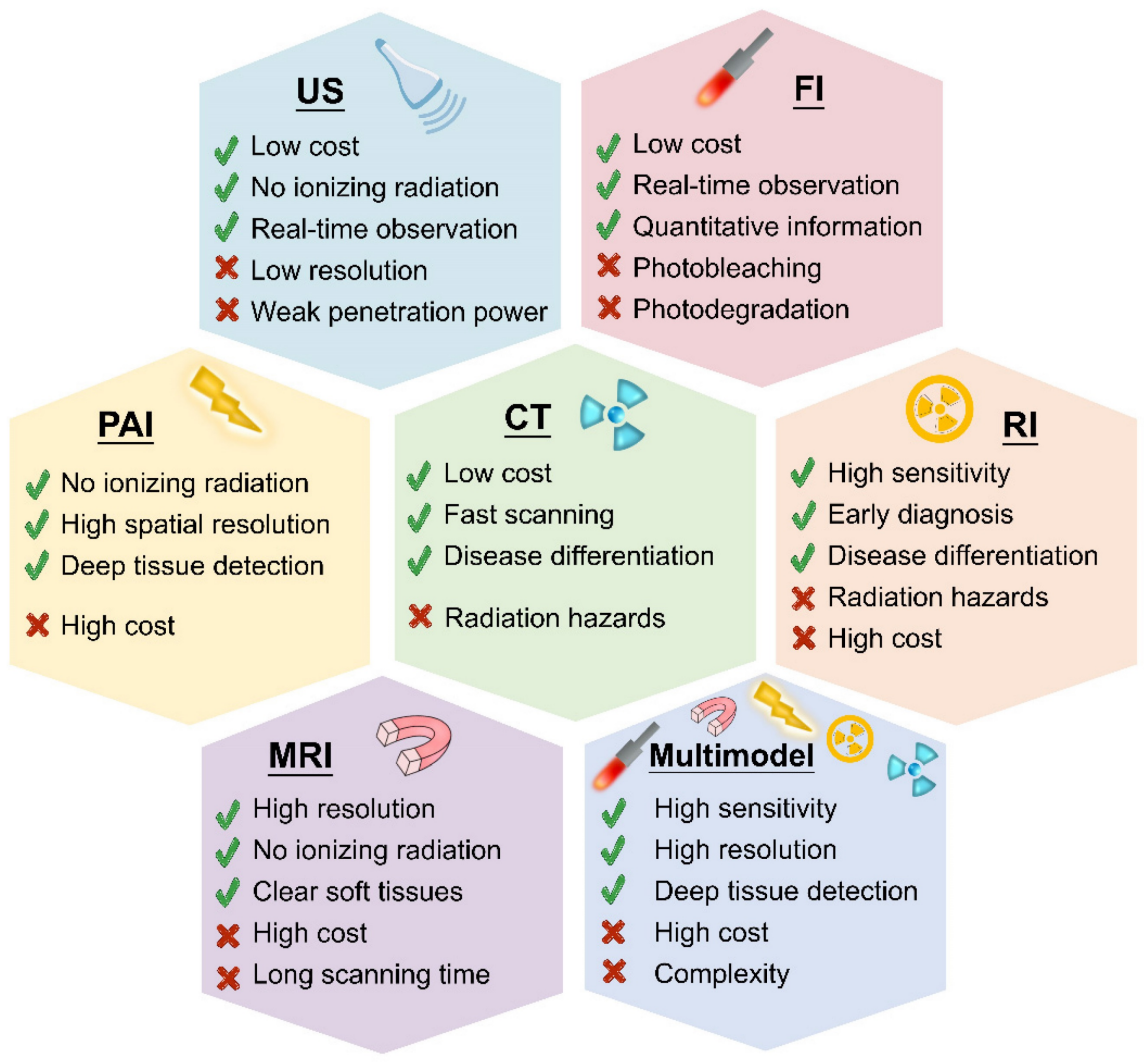
Schematic illustration of strengths and weaknesses of US, FI, PAI, RI, CT, MRI and multimodal imaging.

Several imaging methods are available for inflammation diagnosis, including US 102, FI 103, PAI 104, CT 105, RI 106, MRI 107 and multimodal imaging 108. Figure 4 compares these diagnostic approaches, highlighting their major advantages and disadvantages.

However, the diagnostic agents used in these methods still face some challenges, such as poor inflammation targeting, insufficient accumulation at inflammation sites, and rapid clearance by blood circulation, which affect the accuracy of inflammation diagnosis 109. Therefore, there is a growing interest in combining nanotechnology and conventional imaging technology for inflammation detection, as nanotechnology can overcome some of these limitations and enhance the performance of imaging modalities.

#### US nanoprobes

US imaging is a widely used diagnostic modality for clinical applications, as it harnesses high-frequency sound waves that create echoes when interacting with tissues 110. These echoes detected by ultrasonic sensors are meticulously analyzed for their amplitude and time, forming the basis for generating real-time detailed images 110. To enhance US imaging, contrast agents are often introduced. However, traditional US contrast agents struggle with limited echogenicity and inadequate accumulation at target sites, resulting in reduced imaging resolution 111. To overcome the challenge, nano-based US nanoprobes such as silica NPs and perfluorocarbon-based NPs are introduced 110, 112. For instance, Chudal *et al.* conducted perfluorobutane nanodroplets (NDs) filled with a low-boiling point perfluorobutane as ultrasound nanoprobes to label inflammation-related macrophages 102. The NDs were internalized by macrophages and vaporized into microbubbles by ultrasound, which showed higher contrast-to-noise ratio (CNR), resulting in enhanced US signal and sensitivity. The outcomes also demonstrated that the NDs did not affect the viability and function of the macrophages and that the vaporization could be achieved within the energy limits of a clinical ultrasound scanner. *In vivo*, NDs enabled tracking and labeling of macrophages in rats and achieved visualization of macrophage function 102.

However, US encounters hurdles in depicting bones and lungs owing to the challenges in US wave propagation 110. Therefore, US is often employed with other imaging modalities to boost imaging resolution and diagnostic precision such as FI and PAI 110.

#### FI nanoprobes

FI is an innovative technique for inflammation detection, allowing visualization of dynamic processes and temporal changes in inflammatory sites via intravenous application of a fluorescence dye 113. However, clinical organic dyes, such as indocyanine green (ICG) and methylene blue (MB) often suffer from limited *in vivo* stability, leading to potential issues such as impaired liver or kidney function 113, 114. With the rapid development of nanotechnology, various nanotechnology-driven FI nanoprobes, including QDs-based, aggregation-induced emission (AIE)-based, “turn-on,” and ratiometric fluorescent nanoprobes, have been developed 77, 115. These nanoprobes have demonstrated high optical stability, sensitivity, selectivity and reduced side effects, holding promising potential for accurate diagnosis of inflammation.

QDs have multiple advantages over traditional fluorescent dyes, including high quantum yield, photobleaching resistance, ease of surface modification and tunable emission ranges 90. Leveraging the advances in QDs, QDs-based nanoprobes have been developed for the detection of inflammation 116. For instance, Liu *et al.* successfully integrated elastase-specific peptides, CdSe/ZnS QDs and sulforhodamine B (Rh) into a nanosystem to develop a QDs-based nanoprobe (QDP) for detecting human neutrophil elastase, a common protease in pulmonary inflammation 116. Compared with reported small-molecule and peptide probes, QDP possessed higher sensitivity and lower limit of detection (LOD: 7.15 pM), reduced environmental interference and improved *in vivo* HNE imaging, which helped in distinguishing patients with lung inflammation from healthy individuals 116. Despite multiple advantages, QDs may induce oxidative stress and inflammatory-associated disorders due to the poor biocompatibility and cytotoxicity of heavy metal elements such as Cd and Pb 117. Therefore, the development of emerging organic materials is essential to mitigate the potential toxicity associated with inorganic materials.

AIE-based nanosystems offer a promising platform with unique properties 118. Unlike conventional organic fluorophores that are susceptible to fluorescence turn-off modes by aggregation-caused quenching (ACQ) effect, AIE NPs are highly fluorescent in the aggregated state due to restricted molecular motion, which leads to enhanced radiative decay rate and high photobleaching resistance 119. Furthermore, AIE NPs exhibit high biocompatibility and stability, making them suitable for *in vivo* deep-biological imaging 118. For specific peroxynitrite (ONOO^-^) detection, an inflammation-related species, Xie* et al.* fabricated an AIE-active luminogenic nanoprobe by encapsulating an AIE luminogen, namely tetraphenylethene-dimethylaminoboronic acid (TPE-DMAB), within a lipid-PEG matrix 120. Unlike conventional organic fluorophores, TPE-DMAB demonstrated exceptional sensitivity towards ONOO^-^ (LOD: 54 nM) and desirable chemical stability, resulting in fluorescence enhancement of up to 100-fold upon phenylboronic moiety cleavage by ONOO^-^. *In vivo*, the nanoprobe also facilitated the visualization of ONOO^-^ in a lipopolysaccharide (LPS)-induced inflammation-bearing nude mouse model 120.

**Figure 5:**
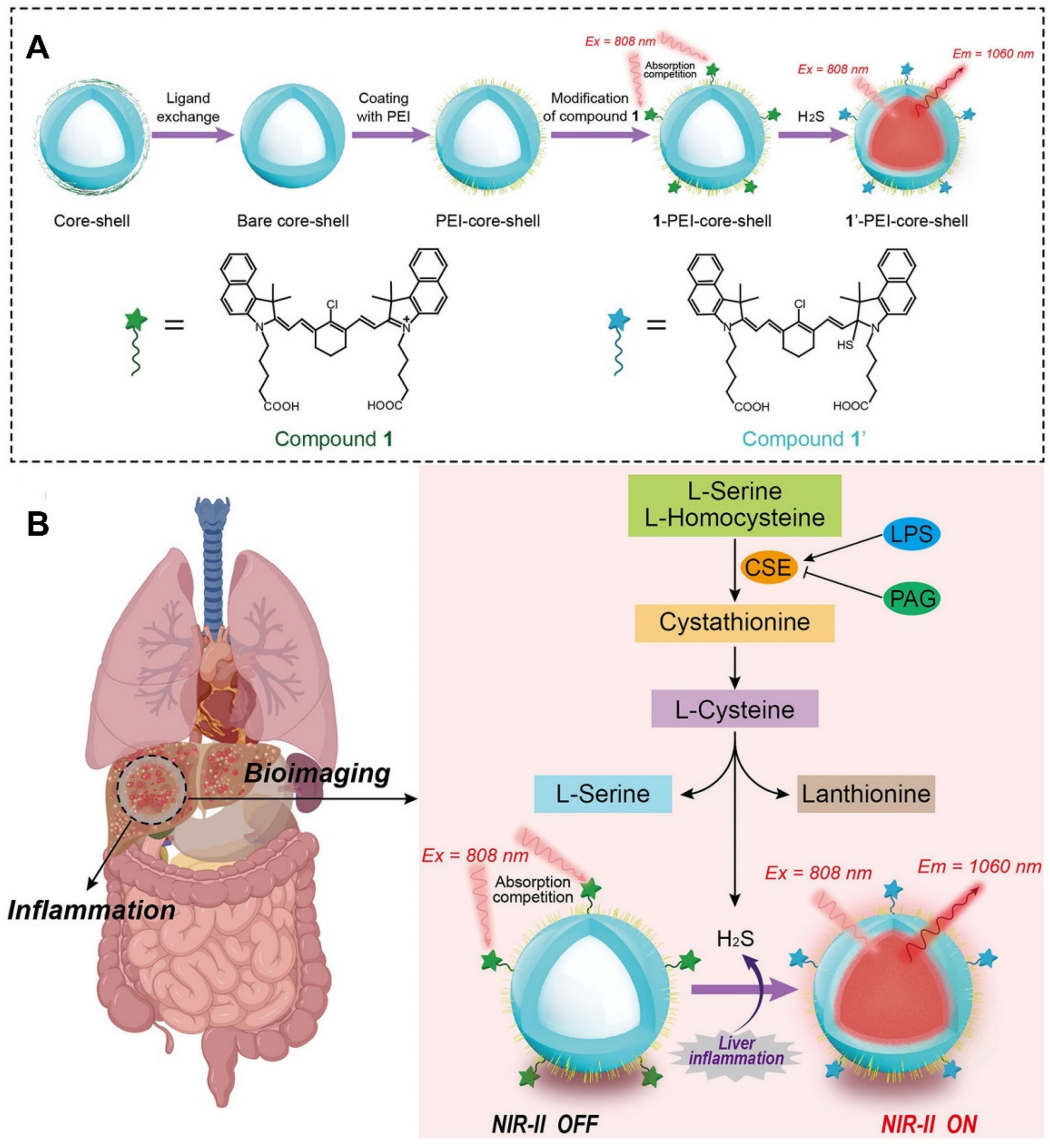
**The structure and imaging mechanism of a NIR-II fluorescent probe. (A)** The synthesis route of 1-PEI-DCNPs. **(B)** The imaging mechanism of 1-PEI-DCNPs. 1-PEI-DCNPs exhibited responsiveness to LPS-induced liver inflammation and high sensitivity to H_2_S, with the ACIE effect completely suppressing their NIR-II luminescent signals at 1060 nm. In the presence of excessive H_2_S generated during LPS-induced liver inflammation, the nucleophilic addition between HS^-^ and the benzpyrole group in compound 1 bleached the absorption of compound 1 at 808 nm. However, the presence of H_2_S within endogenous inflammatory sites activated the fluorescence of 1-PEI-DCNPs at 1060 nm, achieving a "turn-on" luminescence. Adapted with permission, from 124 Copyright 2021 American Chemical Society.

Advancements in “smart” fluorescent nanoprobes that switch between “on” and “off” states have garnered significant attentions 58, 121. Unlike “always-on” fluorescent nanoprobes, “turn-on” fluorescent nanoprobes can be selectively activated by endogenous inflammatory triggers, such as endogenous hydrogen peroxide (H_2_O_2_) and hydrogen sulfide (H_2_S), enhancing both the sensitivity of biosensors and bioimaging resolution 122. Furthermore, near-infrared-II (NIR-II, 900-1700 nm) fluorescent nanoprobes have demonstrated remarkable capabilities in terms of deep-tissue penetration and non-invasive imaging contrast enhancement 123-125. Therefore, integrating both NIR-II fluorescent materials and “turn-on” fluorescent probes into a unified nanosystem might exhibit outstanding sensing capabilities. As shown in Figure 5, Liu* et al.* devised a “turn-on” NIR-II luminescent NPs (1-PEI-DCNPs) by attaching H_2_S-responsive chromophores onto the NaGdF_4_:2%Nd@NaGdF_4_ NPs via polyethyleneimine (PEI) linkers for visual monitoring of overproduced H_2_S *in vivo* 124. By leveraging the absorption competition-induced emission (ACIE) mechanism, the fluorescence emission of 1-PEI-DCNPs at 808 nm was effectively quenched. Nevertheless, the presence of H_2_S within endogenous inflammatory sites could trigger a reaction that activated the fluorescence of 1-PEI-DCNPs at 1060 nm. Notably, given the remarkable penetrability and tissue contrast provided by the NIR-II window, 1-PEI-DCNPs could achieve accurate imaging of liver inflammation and real-time visualization of overproduced H_2_S in a LPS-induced liver inflammation mouse model 124.

Ratiometric FI nanoprobes enable accurate molecular detection and imaging by using signal ratios, overcoming limitations of non-ratiometric nanoprobes such as nanoprobe concentration and background interference 126. Additionally, ratiometric nanoprobes are more accurate and reliable than non-ratiometric ones, providing more information about the analyte, including concentration, location and dynamics 126. For instance,* Pei et al.* developed 3D-printed bioactive glass scaffolds (ErBG@IR808 scaffolds) by incorporating Erbium (Er)-doped NPs (ErNPs) and modifying them with HClO-responsive IR808 fluorophores, enabling real-time monitoring of early-stage inflammation 127. Under 808 nm excitation, IR808 absorption quenched ErBG scaffold emission at 1525 nm via the ACIE mechanism. Increasing HClO reversed this quenching by oxidizing IR808, while the reference signal at 1525 nm remained stable under 980 nm excitation. Furthermore, the dual-excitation ratiometric ErBG@IR808 scaffolds showed a linear correlation with HOCl concentration (LOD: 0.42 μM) at 1525 nm, achieving the visualization of ongoing inflammation during bone repair in a mouse calvarial defect model 127.

#### PAI nanoprobes

Despite the distinct advantages, FI has some defects such as poor tissue penetration and light scattering in biological tissues. By contrast, PAI based on both US and FI, possesses the inherent benefit of high spatial resolution, no ionizing radiation, deep tissue penetration (up to approximately 7 cm) and real-time imaging capabilities, attracting tremendous attention 128. As reported, excessive oxidative stress levels *in vivo* are closely related to chronic inflammatory diseases 129, 130. However, accurate detection of inflammation-related endogenous biomarkers remains a tough task due to the low substrate concentration in physiological environments (∼50 μM) and the limited tissue penetration depth of imaging agents 131. To overcome these obstacles, *Ma et al.* investigated a biocompatible nanoplatform (PLCDP@PMH, 252.7 nm) to load the polymeric photoacoustic probe for AS theranostics 132. The prepared PLCDP@PMH possessed ROS/matrix metalloproteinase (MMP) dual-responsive properties and targeted delivery capabilities. Furthermore, it was also capable of absorbing NIR light and generating robust photoacoustic signals, thus providing high-resolution images of inflamed blood vessels. *In vitro*, PLCDP@PMH demonstrated superior photoacoustic conversion efficiency even at a lower concentration of 5 μg/ml. *In vivo*, PLCDP@PMH facilitated highly contrasting photoacoustic imaging compared to normal tissue, suggesting promising clinical potential for noninvasive AS diagnosis 132.

In spite of significant advancements in PA nanoprobes, accurately measuring inflammation-related biomarkers remains challenging due to their dynamic changes and short lifespans 133, 134. Hence, the design of highly sensitive PA nanoprobes is crucial for monitoring the complex inflammatory microenvironment. Compared to non-ratiometric PAI, ratiometric PAI demonstrates greater accuracy and sensitivity in monitoring excessive ROS in inflamed tissues 104, 135. For example, Ye *et al.* successfully designed an H_2_O_2_-activated ratiometric nanoprobe (Au-Pd@Ag NR) for precise and reliable detection of H_2_O_2_, a biomarker of inflammation, according to the regular change in PA signals 136. Au-Pd@Ag NR consisted of Pd-tipped gold nanorods with an Ag shell that can produce ratiometric PA signals and release Ag ions upon H_2_O_2_ exposure. Notably, the ratiometric PAI (PA_1260_/PA_700_) signals exhibited a direct correlation with H_2_O_2_ concentration, enabling reliable quantification of H_2_O_2_ levels at inflamed sites. Furthermore, the exceptional resolution and deep tissue penetration of Au-Pd@Ag NR enabled precise differentiation between inflamed regions and normal tissues.* In vivo* experiments revealed that Au-Pd@Ag NR could accurately quantify H_2_O_2_ levels in a mouse model of abdominal inflammation and a rabbit model of osteoarthritis (OA) 136.

#### CT nanoprobes

CT imaging is a technique that uses X-rays to scan the human body from different angles and then reconstructs the cross-sectional images of the body using a computer 137. CT visualizes the human skeleton, organs and blood vessels, detailing their structure, size, density and enhancement, which is helpful for diagnosing and staging diseases 137. Compared with MRI, CT scanning offers several benefits such as rapid image acquisition and relatively low costs 138. Despite considerable advancements in CT contrast agents over time, they still have limitations. Specifically, traditional iodinated contrast agents suffer from inadequate targeting, short half-life and severe nephrotoxicity, leading to substantial side effects 139.

In recent years, nanoprobes, specifically AuNPs, have attracted significant interest. AuNPs are ideal CT nanoprobes, which can greatly increase CT contrast with minimal toxicity 140. For example, Yu *et al.* devised a pH-sensitive gold nanotracer (CPP-PSD@Au) functionalized with a cell-penetrating peptide (CPP) and a sulfonamide-based polymer (PSD) to improve the cellular uptake and biocompatibility, leading to prolonged CT monitoring of mesenchymal stem cells (MSCs) in a murine model of idiopathic pulmonary fibrosis (IPF) 141. CPP-PSD@Au exhibited divergent surface charges at pH 7.4 and 5.5, respectively. Upon internalization within endosomes, CPP-PSD@Au progressively aggregated in acidic environments due to PSD protonation and CPP dissociation, promoting substantial cellular retention and enabling long-term tracing of the transplanted stem cells. Employing the CT imaging technique in conjunction with CPP-PSD@Au, the transplanted MSCs were successfully monitored for up to 35 days post-transplantation into the IPF mouse lungs, thus yielding valuable insights into the *in vivo* migration process of MSCs. 141.

Although AuNPs have superior characteristics, such as easy preparation and robust CT contrast, their potential side effects and high costs present challenges for clinical translation 142. With the K-edge of cerium at 40.4 keV, cerium oxide (CeO_2_) NPs have recently emerged as an alternative CT nanoprobe offering substantial x-ray attenuation 142. For instance, *Naha Pratap C. et al*. fabricated dextran-wrapped CeO_2_ NPs (Dex-CeNPs) for diagnosing IBD 142. Dextran is a polysaccharide derived from glucose that could enhance the biocompatibility, stability and water solubility of NPs. Compared with the Food and Drug Administration (FDA)-approved iodinated contrast agents (ICAs), Dex-Ce NPs exhibited greater accumulation at inflamed sites and had a CT contrast enhancement of 1.5 HU per mg/ml in a mouse model of dextran sodium sulfate (DSS)-induced colitis. Remarkably, 97.6% of oral doses were eliminated from the colitis mice within 24 hours, which reduced the risk of toxicity 142.

Bismuth (Bi) NPs have also emerged as promising agents for CT imaging because of their high density and atomic number (Z = 83), which give them strong X-ray attenuation power 143. Furthermore, Bi NPs can be modified with various coatings or functional groups to enhance their biocompatibility, stability, solubility and targeting ability. Bi NPs can also be designed to have different sizes and surface properties, which influence their biodistribution, pharmacokinetics and targeting efficiency 143. For instance, Rabin *et al.* employed a long-circulating bismuth sulfide nanocrystals (BPNPs) coated with polyvinylpyrrolidone (PVP) for CT imaging. Firstly, BPNPs had fivefold better X-ray absorption than iodine, which improved the contrast and resolution of CT images. Furthermore, BPNPs could enable the detection of small lymph nodes (< 1 mm) and liver lesions (<0.5 mm) in mice, which were difficult to image with ICAs. Additionally, they possessed a prolonged circulation times (> 2h)* in vivo* for targeted microvasculature imaging, holding significant potential for broadening the applications of X-ray CT 144. Bi NPs can also provide higher CT values at lower doses and radiation parameters. For example, Tarighatnia *et al.* utilized diethylenetriaminepentaacetic acid (DTPA) as the chelating agent to synthesize a small-molecular bismuth chelate (Bi-DTPA) with high X-ray attenuation and low toxicity 145. Compared with commercial iodine-based contrast agents, Bi-NPs demonstrated higher CT values with a 13-fold increase in CNR. Moreover, MUC-16 aptamer-targeted Bi NPs demonstrated a 6-fold increase in X-ray attenuation over non-targeted Bi-NPs *in vitro* 145. Also, bismuth NPs can be used for dual-modality imaging, such as combining CT with single photon emission computed tomography (SPECT). For instance, Kevadiya *et al.* synthesized rilpivirine (RPV) loaded bismuth sulfide NPs (BiSNRs) and labeled them with lutetium-177 (LuBSNRs) to enable both SPECT and CT imaging 146.

#### RI nanoprobes

RI, also known as nuclear medicine imaging, is the earliest and most extensively used molecular imaging technique in clinical applications 147. It uses molecular probes labeled with radioactive nuclides to detect their distribution and metabolism in the body, reflecting the location, degree and activity of inflammation. RI mainly includes SPECT and positron emission tomography (PET). SPECT relies on gamma-ray-emitting tracers to obtain detailed three-dimensional images, while PET utilizes positron-emitting radioactive tracers, commonly ^18^F-FDG, to highlight areas with high metabolic activity. 147. By utilizing these methods, nuclear medicine provides valuable insights into physiological processes, enhancing our understanding of inflammation.

Despite high sensitivity of PET and SPECT, the range of medical contrast media accessible to clinicians remains constrained by ionizing radiation risk and low spatial resolution 106. More importantly, the half-lives of radiopharmaceuticals (typically a few hours) also restrict quality control procedures 147. NPs represent a promising approach enabling advanced and highly specialized contrast media. Their unique properties allow loading diverse radioactive tracers via various synthesis methods, enhancing targeting capabilities and specificity 106. For instance, Senders *et al.* employed a high-density lipoprotein-derived nanotracer labelled with zirconium-89 (^89^Zr) to allow PET imaging and track the systemic dynamics of leukocytes in atherosclerotic mice 148. The experimental findings demonstrated a significantly elevated PET signal in inflamed sites compared to the background 148. Zhang* et al.* utilized polymer NPs doped with FDA approved diagnostic radioisotope technetium-99m (^99m^Tc) for SPECT imaging of RA 149. ^99m^Tc had low toxicity and fast clearance from the body, which enhanced NPs' biocompatibility and safety. *In vivo* SPECT imaging results demonstrated that ^99m^Tc-NPs accumulated in RA mice, showing the effective and targeted delivery of NPs 149.

PET and SPECT boast distinct advantages over alternative imaging modalities due to their exceptional sensitivity. However, the challenge lies in their constrained spatial resolution, necessitating the co-registration with CT or MRI to achieve accurate diagnosis 148. Additionally, with the advances in nanotechnology, the use of combined PET or SPECT and CT will also broaden the scope of the imaging modality and reduce exposure to ionizing radiation, promoting clinical translation of SPECT and PET techniques 147.

#### MRI nanoprobes

MRI is a valuable tool for the non-invasive diagnostic imaging of inflammation-related diseases by providing high-resolution soft tissue images 150. MRI is a technique that uses a strong magnetic field and radio waves to create detailed images of the organs and tissues in the body 151. The fundamental MRI parameters, T_1_ and T_2_ relaxation times, describe the recovery and decay of magnetization in tissues after being disrupted by radiofrequency (RF) pulses 151. Shorter T1 values in tissues accelerate longitudinal magnetization recovery, resulting in brighter T1-weighted images, while longer T2 values indicate slower decay, leading to darker images 152. Typically, contrast agents employed in MRI are primarily paramagnetic agents such as gadolinium (Gd) or manganese (Mn), and superparamagnetic agents such as superparamagnetic iron oxide particles (SPIO) 152. Paramagnetic agents mainly shorten T_1_ relaxation time, thereby enhancing signal intensity on T_1_-weighted images 153. On the other hand, superparamagnetic agents mainly reduce T_2_ relaxation time, displaying T_2_ hypo signal. However, these contrast agents exhibit limitations such as suboptimal biocompatibility and targeting inefficiencies.

In recent years, nanotechnology-assisted modification of contrast agents has shown immense promise to mitigate these issues. For example, He *et al.* engineered a brain-targeted nanoconstruct (aAβ-BTRA-NC) activated by ROS for detecting and monitoring AD progression 154. aAβ-BTRA-NC consisted of manganese oxide NPs (MnO_2_) for MRI imaging, a polymer/lipid core for stability and biocompatibility as well as an anti-amyloid-beta (Aβ) antibody for targeting. Due to the presence of targeting moieties, aAβ-BTRA-NC effectively penetrated the BBB and bound to Aβ plaques in the brain. Upon exposure to ROS, aAβ-BTRA-NC facilitated the local release of Mn^2+^, amplifying T_1_-weighted MR signals in the cerebrospinal fluid (CSF) by a factor of 1.51-2.24. In an AD mouse model, aAβ-BTRA-NC exhibited exceptional sensitivity (89%) and specificity (100%) in detecting early-stage AD 154.

Since the approval for clinical application by the FDA, SPIO NPs with small particle sizes have attracted tremendous scientific interest due to their exceptional chemical, magnetic and biocompatible properties 155. Based on the selective aggregation-enhanced T_2_ effect of SPIO NPs, *Tang et al.* designed platelet-mimetic NPs (PTNPs) incorporating SPIO to monitor activated neutrophils in ischemic stroke 156. Compared with PTNP control groups, SPIO-PTNP groups demonstrated superior biocompatibility, enhanced targeting efficiency and improved MRI contrast, enabling real-time monitoring of inflammatory progression 156.

Despite the advancements made with SPIO NPs, they encounter magnetization saturation at around 1.5 T, limiting MRI improvements at higher magnetic fields 157. Dysprosium (Dy^3+^) has been identified for its substantial magnetic moment, short relaxation time and magnetic saturation surpassing 21 T via a Curie mechanism 157. To augment the T_2_ relaxation rate, PAA-modified ultrasmall IO/Dy oxide NPs (IO-DyO NPs) were designed for precise liver fibrosis imaging 157. Within IO-DyO NPs, IO showed magnetic characteristics conducive to MRI, while DyO served as a contrast agent to amplify the imaging signal. Specifically targeting the fibrotic regions in the liver, IO-DyO NPs boosted the sensitivity and specificity of MRI for detecting and characterizing liver fibrosis. In comparison to conventional SPIO NPs, IO-DyO NPs exhibited a threefold increase in r_2_ (1/T_2_ s^-1^) relaxivity, which was particularly evident under a 9.4 T MRI system. Furthermore, IO-DyO NPs significantly improved the spatial and temporal resolution of liver imaging, enabling accurate discrimination of fibrotic liver tissues and facilitating clear staging of the clinically consequential early and moderate liver fibrosis 157.

#### Multimodal imaging nanoprobes

Inflammation is a complex biological process that involves various tissues and cells in response to injury or infection. Imaging techniques can help to detect, monitor and characterize inflammation in different organs and systems. However, no single imaging modality can provide all the information needed for a comprehensive assessment of inflammation, such as high resolution, deep tissue penetration, high sensitivity, fast imaging and low toxicity 158. Therefore, multiple imaging modalities are often combined to overcome the limitations of each individual technique and to obtain complementary information. Nanotechnology has sparked interest in combining various imaging methods on a nanoplatform. This integration harnesses their individual strengths, offering detailed information, enhanced sensitivity and specificity, improved spatial and temporal resolution and synergistic data interpretation 108. However, this approach comes with challenges, such as technical complexities and the high costs 108.

As previously discussed, the PA nanoprobe demonstrates high spatial resolution and deep tissue penetrability, exhibiting considerable potential as a ROS imaging modality. In addition, CT is considered a powerful technique that provides images with high spatial and temporal resolution. Consequently, combining PA and CT on a nanoprobe presents a solution for ROS imaging at inflamed sites 159. For example, Bouche M* et al.* devised a ROS-responsive hybrid nanoprobe (PPB NP) for dual CT/PA imaging by incorporating small AuNPs and polyphosphazene derivatives (PPB) nanogels 159. Under ROS conditions, PPB NP selectively degraded, triggering a 73% reduction in PA signals, with CT signals remaining stable. This contrast between stable CT and diminishing PA signals effectively distinguished ROS-overproducing macrophages from non-inflamed ones, enabling the detection of endogenous ROS in inflamed macrophages 159. Similarly, Dai *et al.* developed a type of NPs based on Gd-doped Prussian blue (GPB) for MRI/fluorescence dual-modality imaging, offering complementary information and improving the accuracy and sensitivity of AS plaque detection 160.

Beyond the combination of two imaging modalities, more imaging agents can also be utilized for accurate diagnosis. For instance, Gong* et al.* constructed a nanozyme-based ratio-metric nanoprobe (FeWOX NS) by co-loading 3,3,5,5-tetramethylbenzidine (TMB) and IR780 dye on FeWOX nanosheets (NSs) for PA/MRI/CT imaging of H_2_O_2_-related inflammation 128. FeWOX NS exhibited high PA sensitivity (LOD: 0.5 µM) for H_2_O_2_ detection and the ratio-metric PA signal could distinguish the different levels of H_2_O_2_ in tumor and inflammation tissues. FeWOX NS also served as CT and MRI nanoprobes due to their high X-ray and MR contrast abilities, surpassing commercial iodine and Gd-based agents, respectively. Importantly, owing to their inherent biodegradability, FeWOX NSs could be cleared out from the body without any significant biotoxicity 128.

### Summary

In summary, nanosensors and nanoprobes have played crucial roles in early inflammation detection and precise monitoring. Nanosensors have emerged with the benefits of low cost, high efficiency, sensitivity and specificity. Some of them have entered clinical trials, such as a nanosensor array for multiple sclerosis diagnosis (NCT04074629) 3. Alongside accuracy and sensitivity, POCT platforms also emerge as a potential solution to meet the need for affordable nano biosensors with rapid tests and user-friendly panels.

Although biosensors can identify the presence of inflammation, they do not offer information about the exact location. Imaging methods show their advantages in visualizing tissue at both the structural and functional levels. In recent years, many nanoprobes have been developed for inflammation imaging and some of them are summarized in Table 2. Nanoprobes hold great promise in enhancing imaging modalities for clinical imaging, including US, CT, RI and MRI. In US, nanoprobes enhance acoustic signals for improved visualization of vascular structures. For CT, nanoprobes enhance X-ray attenuation, improving contrast in anatomical structures. In RI, nanoprobes act as effective tracers for precise detection of functional and molecular changes. In MRI, nanoprobes amplify magnetic properties, improving tissue and organ visibility. However, each imaging modality still has its own limitations, as shown in Figure 4. The choice depends on clinical requirements, the nature of information needed and considerations like radiation exposure and cost, collectively contributing to a comprehensive diagnostic toolkit.

Nanoprobes also show promise in preclinical studies for FI and PAI that can provide high-resolution and real-time images, although not yet applied clinically. Firstly, NPs offer advantages over clinical organic dyes such as ICG and MB, with higher stability, lower toxicity, and increased sensitivity and selectivity 161. In PAI, NPs also serve as contrast agents, delivering real-time images with deep tissue penetration. They can target inflammatory cells or detect changes in inflammatory biomarkers, such as excessive oxidative stress levels, for imaging inflammation. Finally, FI or PAI nanoprobes can integrate with other imaging modalities to provide complementary information and enhance the diagnostic accuracy. Despite these capabilities, challenges remain for clinical translation of nanoprobes in FI and PAI, including the imaging parameter optimization and standardization of imaging protocols. Therefore, further research and development are essential to overcome these challenges and to realize the full potential of FI and PAI for inflammation diagnosis in the clinic.

## Anti-inflammatory nanotherapeutics

**Figure 6:**
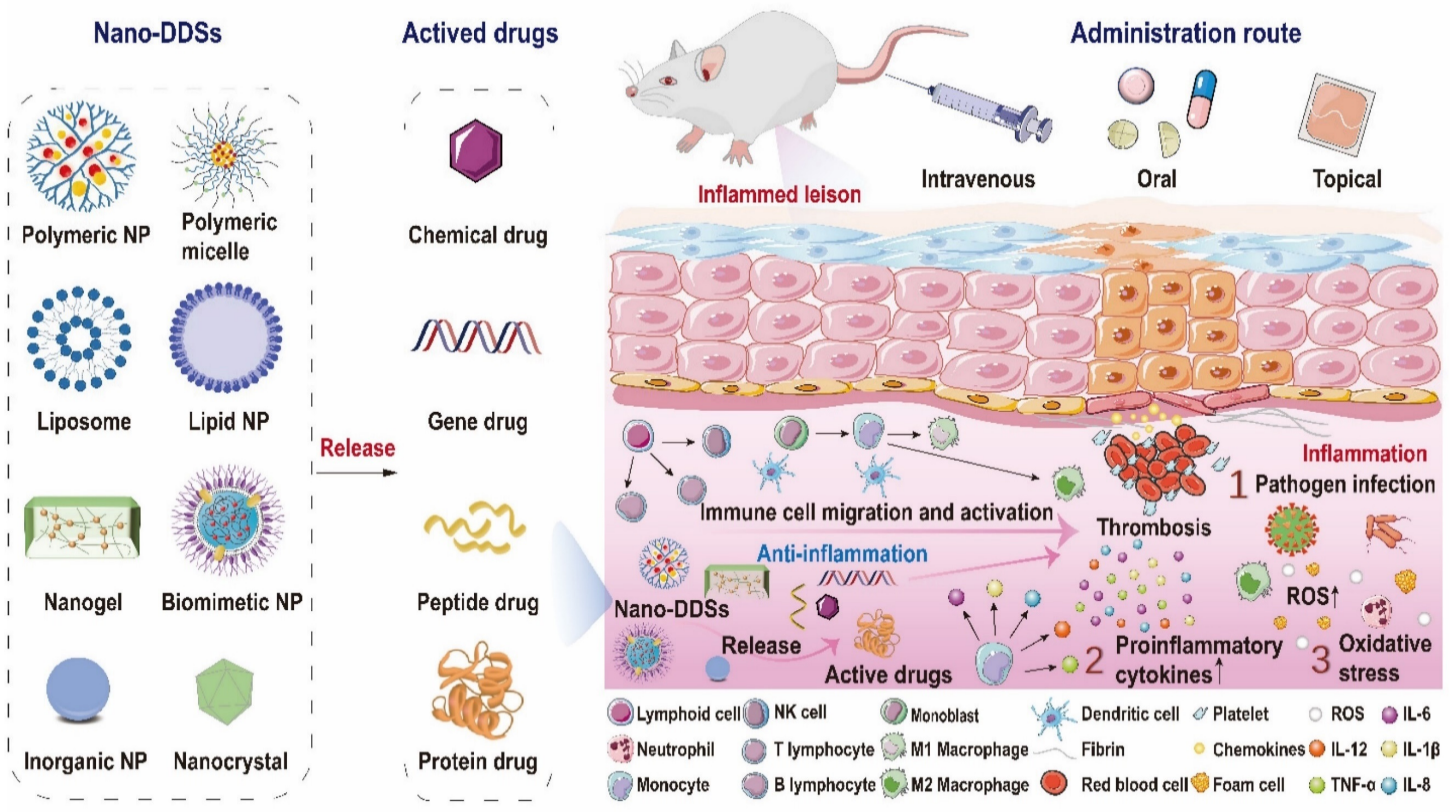
Schematic representation of the complex inflammatory microenvironment and the therapeutic nano-DDSs employed for the regulation of inflammation. The inflammatory microenvironment encompasses invasive pathogens, impaired cells, infiltrating immune cells and a plethora of pro-inflammatory molecules, with elevated levels of oxidative stress and intravascular thrombosis sometimes. Versatile nano-DDSs can be applied to deliver chemical, gene, peptide or protein drugs to the targeted sites to achieve satisfactory therapeutic outcomes.

Over the decades, although multiple anti-inflammatory therapeutic agents have been widely developed and applied in the clinic, there are still several existing limitations for traditional DDSs including off-target biodistribution in the body, potential safety concerns of virus vectors and rapid clearance from the blood. Consequently, ongoing efforts have been put forward to develop novel DDSs to achieve satisfactory therapeutic outcomes 172. Among them, nano-DDSs, characterized by inherent advantages such as site-specific drug delivery 55, 173, favorable bioavailability 174, 175 and time-controlled drug release 176, 177, have emerged as a promising therapeutic platform for the treatment of inflammation-related diseases (Figure 6). In this section, several emerging nano-DDSs for delivering chemical, gene and protein drugs as well as self-therapeutic NPs are mainly discussed.

### Chemical drug nanotherapeutics

Recently, advanced nano-DDSs including lipid-based NPs, polymeric NPs, polymeric nanomicelles, nanogels, biomimetic nanomedicines and other nanoformulations, have opened up novel possibilities for improving the effectiveness of anti-inflammatory treatment while minimizing adverse effects 43, 178, 179. A comprehensive summary of recent nanotherapeutics based on small-molecule chemical drugs is provided in Table 3.

Lipid-based NPs (LNPs) have long been employed as promising nano-vehicles for the delivery of therapeutic agents 180. Comprising lipids such as phospholipids or solid lipids, LNPs demonstrate outstanding biocompatibility and stability 181. LNPs enable the encapsulation of hydrophobic drugs within lipid bilayers and solubilization of hydrophilic drugs in aqueous cores 180. Currently, significant attention is also focused on designing targeted nanomedicine by grafting specific ligands, such as peptides, onto the surface of LNPs to achieve active targeting 180. For instance, Wu *et al.* devised peptides coupled celastrol (CLT)-phospholipid LNPs (PC-PLNs, 114.0 nm, 11.9 mV) to efficiently deliver CLT, a natural anti-inflammatory compound, to damaged endothelial cells and podocytes in the glomerulus for chronic kidney disease (CKD) treatment 182. The PC-PLNs were prepared by self-assembly of CLT, phospholipids and a peptide (GLP) that can specifically bind to the glomerular basement membrane and facilitate the transcytosis of the NPs across the endothelial cells 182. PC-PLNs demonstrated a robust therapeutic effect by selectively releasing CLT to the podocytes, which are the main target cells for CKD treatment, leading to inflammation reduction via nitric oxide upregulation and vascular cell adhesion molecule-1 (VCAM-1) expression inhibition. *In vivo*, PC-PLN treatment resulted in significant amelioration of CKD progression as well as reduced endothelial damage and CLT toxicity in a rat model of CKD induced by adenine 182.

Polymeric nanomedicines (PNs) also form a significant category of nano-DDS, including both dendrimers and polymer-drug conjugate 183. PNs, characterized by their notable attributes of superior drug loading capacity, precise targeting to specific sites and regulated drug release, are emerging as an alternative approach for managing inflammatory diseases 184. Recently, *Shen et al.* constructed a polymer-based nano-DDS (PPP-ACPP, 243 nm, 1.0 eV) to load the clinical anti-inflammatory drug etanercept (ET) for the treatment of spinal cord injury 54.

PPP-ACPP was composed of a biocompatible polymer (PLGA-PEI-mPEG, or PPP) and an MMP-responsive molecule (activated cell-penetrating peptides, or ACPP), which could enhance the penetration of the NPs across the blood spinal cord barrier and the accumulation at the injured site. *In vivo*, ET@PPP-ACPP NPs accumulated at the lesion tissue and targeted inflammatory sites due to the activation of cell-penetrating ACPP by MMP-2 and MMP-9. Notably, ET within these particles moderated macrophage polarization from M1 to M2 phenotype, significantly reducing inflammation in the spinal cord injury mouse model, thereby protecting neurons and enhancing locomotor recovery. Such an activated target-based nanocarrier demonstrated considerable potential in improving the delivery efficiency of anti-inflammatory drugs 54.

Polymeric nanomicelles (PNMs) have attracted attention due to their cost-effectiveness, ease of preparation and reduced side effects 185. PNMs consist of two functional components: a hydrophobic inner core and a hydrophilic outer shell 186. The inner core is responsible for encapsulating hydrophobic drugs and maintaining the stability of nanomicelles. Additionally, the outer shell improves pharmacokinetic properties of nanodrugs, such as extended circulation time 185. For example, Akshay Vyawahare *et al.* developed 9-aminoacridine (9AA)-encapsulated nanomicelles (9AA-NM, 190.0 nm, -20.6 mV) for the treatment of RA 187. First, a promising amphiphilic block copolymer, methoxy polyethylene glycol polycaprolactone block copolymer (mPEG-b-PCL) was synthesized and then conjugated with the hydrophilic caffeic acid (CA). The 9AA drug, an FDA-approved anti-inflammatory drug, was eventually incorporated into the PNMs to activate the nuclear receptor subfamily 4 group A member (NR4A1), which has anti-inflammatory and protective effects in RA. Unlike nano-DDS with poor efficacy, the synthesized nanomicelles exhibited good biocompatibility and low toxicity, as they did not cause any adverse effects on the liver, kidney, or blood of the mice. In a rat model of RA, rats treated with 9AA-NMs demonstrated alleviated arthritic symptoms, along with a reduction in RA-associated inflammation 187.

**Figure 7:**
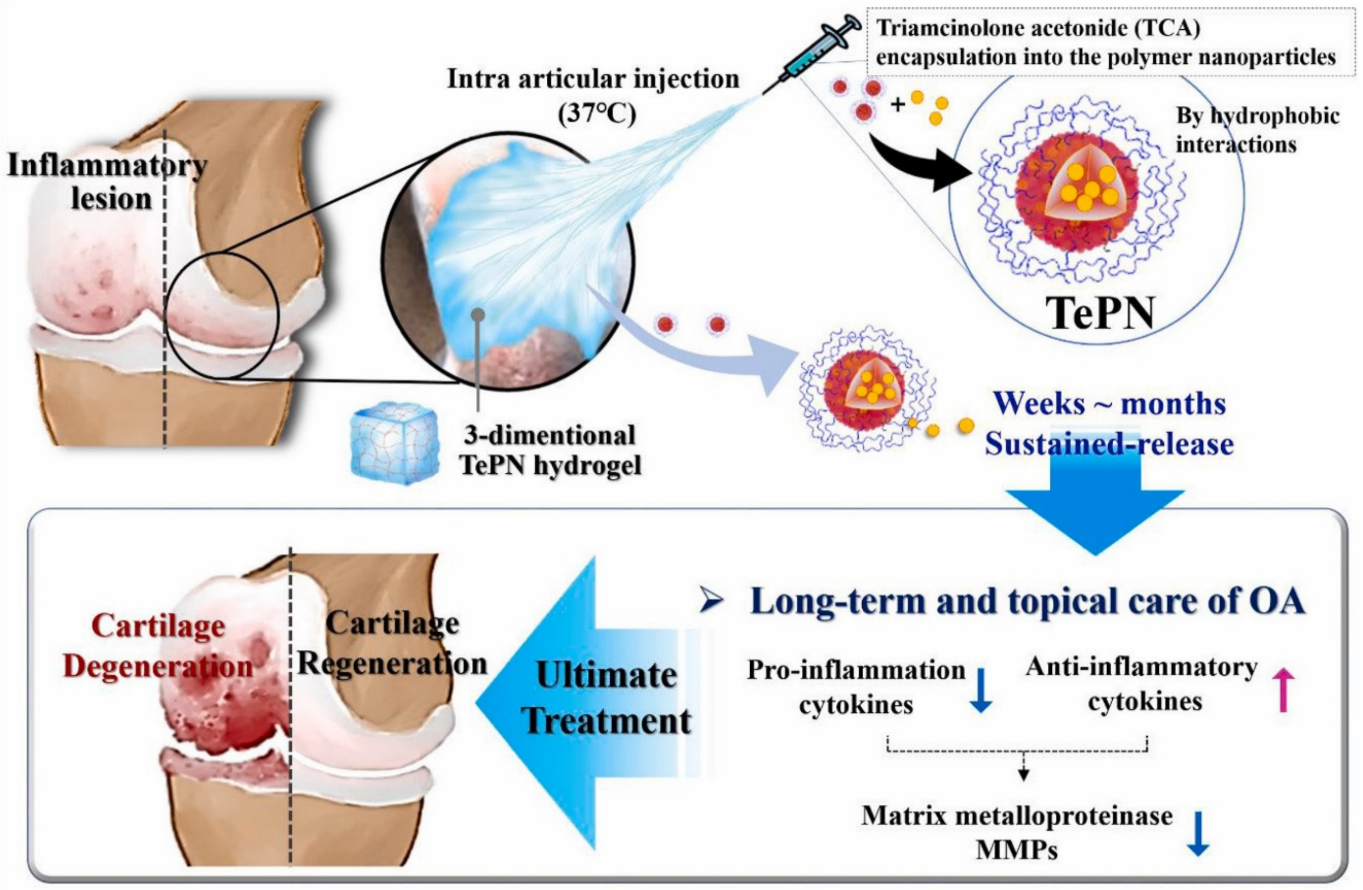
A one-time intra-articular injection of the TePN hydrogel system for long-term OA treatment. First, water-insoluble TCA was loaded into the hydrophobic core of PN by hydrophobic self-assembly, forming TePNs. Upon intra-articular administration at body temperature, the NPs formed into a 3D hydrogel structure and released TePNs for several months. TCA reduced the levels of MMP and pro-inflammatory cytokines, and increased those of anti-inflammatory cytokines in cartilages, treating OA effectively and safely *in vitro* and *in vivo*. Adapted with permission, from 190 Copyright 2022 Elsevier.

Nanogels have a physically three-dimensional (3D) hydrogel structure with a particle size ranging from 20 to 250 nm 188. Unlike conventional NPs, nanogel-based nano-vectors can form size-switchable 3D hydrogel networks by incorporating surfactants such as Tween^®^, Span^®^, polysorbic acid and sodium cholate into nanogels, providing a versatile platform for drug encapsulation and release due to their high water content and porosity of nanogels 189. For example, triamcinolone acetonide (TAC) has fast clearance and adverse effects via intra-articular injection or oral administration 190. To provide long-term effective therapy, Seo *et al.* developed injectable triamcinolone acetonide (TCA)-encapsulated polymeric hydrogel NPs (TePNs, -4.0 mV) for the sustainable and effective treatment of OA 190 (Figure 7). Polymeric NPs were successfully loaded with TCA via the interactions between the hydrophobic segments of the amphiphilic polymer and the hydrophobic TCA. Upon intra-articular administration at body temperature, the NPs transformed into a 3D hydrogel structure. *In vitro*, TePNs achieved long-term release for 6 weeks by inhibiting the expression of MMP. *In vivo*, the TePNs exhibited sustained anti-inflammatory effects without any skin irritation or systemic adverse effects in an early stage of rat model of OA 190.

Biomimetic NPs are a nascent category within the field of nanomedicine by emulating the properties and behaviors of native cells or exosomes, thereby enhancing biocompatibility and targeted drug delivery of NPs 191-193. Among them, the red blood cell (RBC) membrane could help NPs evade the phagocytic system and achieve a prolonged circulation half-life in the circulation (about 120 days), which has gained attention for the development of nanocarriers 191, 194. Wang* et al.* employed RBC membrane-coated biomimetic rapamycin-loaded PLGA nanocomplexes (97.4 nm, -28.7 mV) for AS treatment. The application of RBC membranes led to reduced macrophage-mediated phagocytosis *in vivo*, increased nanoparticle accumulation at atherosclerotic plaques, and decelerated AS progression 195. Inflammatory effector cells, which exert substantial influence on the initiation and advancement of diverse inflammatory diseases, have been harnessed as biomimetic carriers for administering small-molecule pharmaceutical agents 196. For instance, Song *et al.* synthesized macrophage cell membrane-camouflaged NPs (M-EC, 210.0 nm) containing epigallocatechin gallate (EGCG) and Ce^4+^ 197. The macrophage cell membranes were beneficial to enhance the biocompatibility, immune evasion and targeting ability of the NPs to inflamed joints. In a mouse model of collagen-induced arthritis (CIA), M-EC resembling natural antioxidant enzyme sites, showed significant therapeutic effects by reducing the levels of pro-inflammatory cytokines, oxidative stress and cartilage erosion in the joints 197. Combining anti-inflammatory and immune-evasive properties of M2 macrophages, the biocompatibility and long circulation of erythrocytes may produce desirable therapeutic efficacy. Most recently, Chen *et al.* developed a novel type of biomimetic nanosized liposome (USM[H]L, 139.2 nm, -13.1 mV) that was coated with hybrid membranes derived from M2 macrophages and erythrocytes 198. USM[H]L were loaded with uricase and methotrexate, which could synergistically degrade uric acid, scavenge H_2_O_2_, produce photothermal effects and modulate immune responses. Upon being injected into a rat model of gouty arthritis (GA), USM[H]L showed enhanced therapeutic effects by reducing uric acid levels, ankle swelling, claw curling, inflammatory cytokines and oxidative stress, while increasing anti-inflammatory cytokines and reprogramming M1 macrophages to M2 phenotype. Notably, USM[H]L also exhibited low immunogenicity and toxicity, as well as high stability and targeting ability, compared to free uricase or other formulations, representing a promising strategy for the treatment of GA and other inflammatory-related diseases 198.

### Gene drug nanotherapeutics

The era of gene therapy has emerged with the rapid development of genomics and gene technology. Gene therapy, compared to chemical drugs, may offer a more target-specific and safe therapy by inhibiting gene expression or cleaving abnormal messenger RNA (mRNA) 208. Gene drugs mainly consist of small interfering RNA (siRNA), mRNA and plasmid DNA (pDNA). However, naked gene drugs are highly susceptible to degradation by nucleases 209.

Virus vectors are effective for delivering genes into cells, but they also have several disadvantages, including safety concerns, immune responses, complex preparation and limited drug loading capabilities. Consequently, there is an urgent need for suitable carriers to protect gene drugs and overcome these challenges. In the case of non-viral vehicles, such as cationic LNPs, cationic polymers and other NPs have been extensively explored for gene-based nanomedicines. These carriers present a myriad of advantages, including sample preparation, high safety, easy modification and superior stability, making them ideal for gene therapy 210.

As a type of cationic LNPs, cationic liposomes present a versatile nano-DDS for gene delivery due to their capability to bind to negatively charged nucleic acids 211. Notably, multiple reports have demonstrated that cationic liposomes can fuse with cellular plasma membranes, facilitating the direct release of cargos into the cytoplasm and thereby improving the speed and efficiency of drug delivery 212. Furthermore, the downregulation of pro-inflammatory cytokines, particularly TNF-α, may assume a pivotal role in inflammation treatment 213. Recently, LNPs loaded with either siRNA or mRNA have emerged as a promising strategy for modulating the immune system and treating inflammation. For example, Guo *et al.* developed a ROS-responsive cationic liposome (ZnDPA-R, ∼100 nm) to deliver TNF-α siRNA for colitis treatment by taking thioketal as a linker to bind the hydrophobic tails and the metal complex headgroup 213. The targeted liposomes were prepared by spraying the organic phase containing the lipid and the siRNA onto the aqueous phase containing the edge activators, which could overcome the limitations of conventional therapies for colitis, such as low bioavailability, poor specificity, and severe side effects. *In vivo*, ZnDPA-R could accumulate in the intestine, respond to the high ROS level by breaking the thioketal bond, silence the expression of TNF-α and related inflammatory factors, as well as alleviate the inflammatory symptoms, ultimately ameliorating colitis symptoms in a mouse model of colitis 213.

In addition to enhancing intracellular delivery via cell membrane fusion, LNPs can also be modified with various molecules to target specific tissues or cells with gene drugs 214. For instance, Tao *et al.* rationally designed S2P-conjugated lipid-based NPs (S2P_50_-siCamk2g NPs) to specifically deliver siRNA to atherosclerotic lesional macrophages 214. S2P_50_-siCamk2g NPs were prepared by encapsulating siRNA/cationic lipid complexes into PLGA polymer, followed by coating DSPE-PEG and plaque macrophage-targeting peptides (S2P) on the surface. By integrating targeting peptides onto their surface, S2P_50_-siCamk2g NPs demonstrated targeting capability in both cultured macrophages and* in vivo*. Owing to their unique attributes, such as prolonged circulation, selective targeting, superior plaque permeation and effective gene silencing, S2P_50_-siCamk2g NPs played an important role in silencing plaque-destabilizing gene (CaMKII) expression in macrophages as well as promoting plaque stabilization both *in vitro* and *in vivo* 214.

Similarly, Ralvenius *et al.* developed a novel microglia LNP (MG-LNP, 60-80 nm) to efficiently deliver RNA to microglia for treating neuroinflammation and neurodegeneration 215. PU.1 is a potential therapeutic target for reducing neuroinflammation in AD and other neurological disorders. Therefore, reducing PU.1 expression in microglia may have beneficial effects on reducing neuroinflammation and improving cognitive function in AD. MG-LNP was optimized for microglia delivery by screening different lipid compositions and ratios (DLin-MC3-DMA: 1,2-distearoyl-sn-glycero-3-phosphocholine: cholesterol: PEG = 50:10:38.5:1.5). Instead of systemic injection, MG-LNP containing anti-PU.1 siRNA exhibited low toxicity and enhanced delivery to activated microglia via a local intracerebroventricular injection into the cerebrospinal fluid, which bypassed the BBB. *In vivo*, MG-LNP-mediated delivery of anti-PU.1 siRNA successfully exhibited efficiency at transfecting microglia and reduced PU.1 expression in a mouse model of neuroinflammatory conditions, offering a promising avenue for targeted gene therapies against neuroinflammation 215.

Cationic lipid-like materials are also useful in nanomedicine because they can enhance the delivery and expression of mRNA in target cells 216. Gao* et al.* developed a novel mRNA nanomedicine (*IL-10* mRNA@M-HNPs, 122 nm, 2.33 mV) that selectively delivered *IL-10* mRNA to macrophages in AS plaques 216. Targeted nanocarrier consisted of a cationic lipid-like material and a mannose-coated PLGA-PEG that bound to the mannose receptor (CD206) on the surface of macrophages, enhancing the uptake and accumulation of the mRNA nanomedicine in the plaques. The nanomedicine induced the expression of IL-10 in macrophages, promoting their polarization to the M2 anti-inflammatory phenotype. This created a positive feedback loop that modulated plaque inflammation by blocking several pro-inflammatory cytokines such as TNF-α, IL-1β and IL-6 and slowed down AS. The nanomedicine also protected the mRNA from degradation and immunogenicity issues. In a western diet-fed *Ldlr^-/-^* mouse model, the outcomes demonstrated that the selective delivery of *IL-10* mRNA to macrophage-rich plaques and induced IL-10 played important roles in reducing AS 216.

**Figure 8:**
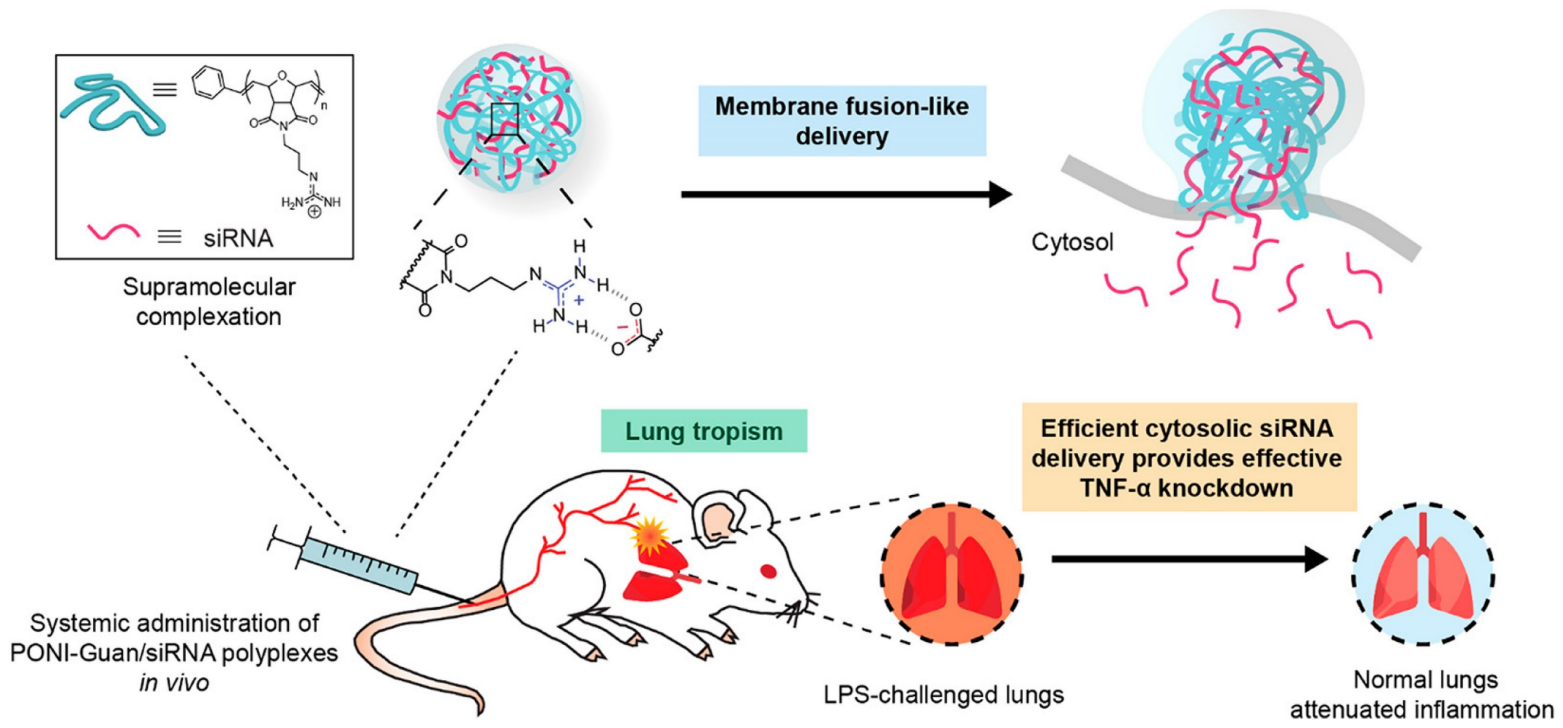
The PONI-Guan/siRNA polyplex self-assembly and its membrane fusion-like siRNA delivery mechanism. Upon systemic administration of PONI-Guan/siRNA polyplexes, the nanocomplexes were preferentially distributed to inflamed lungs with priority and exhibited potent anti-inflammatory activity via efficient TNF-α knockdown by siRNA. Adapted with permission, from 218 Copyright 2023 American Chemical Society.

Functional polymers, characterized by diverse chemical components and topological structures, are being increasingly utilized for the intracellular delivery of nucleic acids 217. Among them, cationic polymers are emerging as ideal nucleic acid drug delivery carriers due to their low immunogenicity and easy structure modification 209. As shown in Figure 8, Jeon* et al.* developed a potent siRNA-based therapeutic approach for the treatment of inflamed lungs by targeting silencing of TNF-α 218. The synthesized cationic polymer, poly (oxanorbornene imide)-guanidinium (PONI-Guan), exhibited the capacity to spontaneously assemble into well-defined nanoscale polyplexes (∼170 nm) in conjunction with siRNA. Notably, this approach achieved efficient silencing (>70%) by using significantly lower siRNA dosages (0.14-0.28 mg/kg) in contrast to those used in current clinical studies, thereby mitigating the risk of off-target effects. Furthermore, the inherent modularity of the PONI system enabled prospective modifications to target additional organs.* In vivo*, PONI-Guan/siRNA polyplexes with a minimal siRNA dosage (0.28 mg/kg), achieved a threefold increase in pulmonary accumulation compared to control mice in an LPS mouse model, resulting in an effective knockdown of serum TNF-α (>80%) 218.

Although classical cationic lipids or cationic polymers enable nucleic acids delivery with high efficiency, they are still suffering from cytotoxicity or immunogenicity 219. Therefore, there is a need for novel noncationic carriers that can deliver nucleic acids safely, efficiently and specifically to the diseased sites. For instance, Bai *et al*. invented a novel three-dimensional spherical noncationic nucleic acid nanostructure (miR-146a-SPIONs, 72.7 nm, -8.2 mV), consisting of a PEG-coated SPION NP core and a nucleic acid shell containing phosphorothioate (PS)-modified microRNA-146a for treating AS 219. Such a nanocarrier demonstrated several advantages. Firstly, miR-146a-SPIONs were non-ionic and did not cause cytotoxicity or immune response. Next, miR-146a-SPIONs naturally targeted the class A scavenger receptor (SR-A) in the atherosclerotic plaque *in vivo*, improving the drug delivery efficiency. Furthermore, miR-146a-SPIONs entered the cell without a transfection agent, released microRNA-146a and suppressed the NF-κB signaling pathway, reducing inflammation and stabilizing the plaques. In a mouse model of apolipoprotein E knockout (ApoE^-/-^), repeated injections of miR-146a-SPIONs showed improved delivery efficiency, reduced plaque size and stabilized plaque morphology without causing severe toxicity, providing a safe and effective method for AS treatment 219.

### Protein or peptide drug nanotherapeutics

Therapeutic proteins and peptide drugs represent new frontiers of drug research and development, as they show better target affinity and safety than chemical drugs 74, 220. However, their clinical efficacy of them is often hindered by several drawbacks, including high plasma clearance rates, short circulation duration, instability, and side effects resulting from frequent administration 221. Thus, the utilization of nanotechnology and pharmaceutical technology to create long-acting, stable and effective formulations holds substantial clinical value 222.

Cytokines, immune cell-secreted proteins acting as intercellular messengers, are crucial for normal physiological functions and serve as therapeutic targets 223. For instance, IL10 has protective properties against atherosclerotic inflammation, but suffers from rapid *in vivo* clearance by proteases 224. To overcome the constraint, Kim *et al.* employed a cyclic arginine-glycine-aspartic acid (cRGD)-conjugated pluronic-based nano-carrier (NC) to encapsulate IL10 and iron oxide NPs for targeted delivery to AS lesions 224. The therapeutic IL-10 was deftly loaded into NC in relatively mild conditions and enabled sustained release, showing equivalent efficacy to free IL10 in inhibiting the generation of pro-inflammatory cytokines and ROS *in vitro*. Of note, the IL10-loaded NC showed vastly improved circulation time, drastically high accumulation at targeted sites and significantly reduced atherosclerotic plaque regression in an apolipoprotein E-knockout mouse model 224.

**Figure 9:**
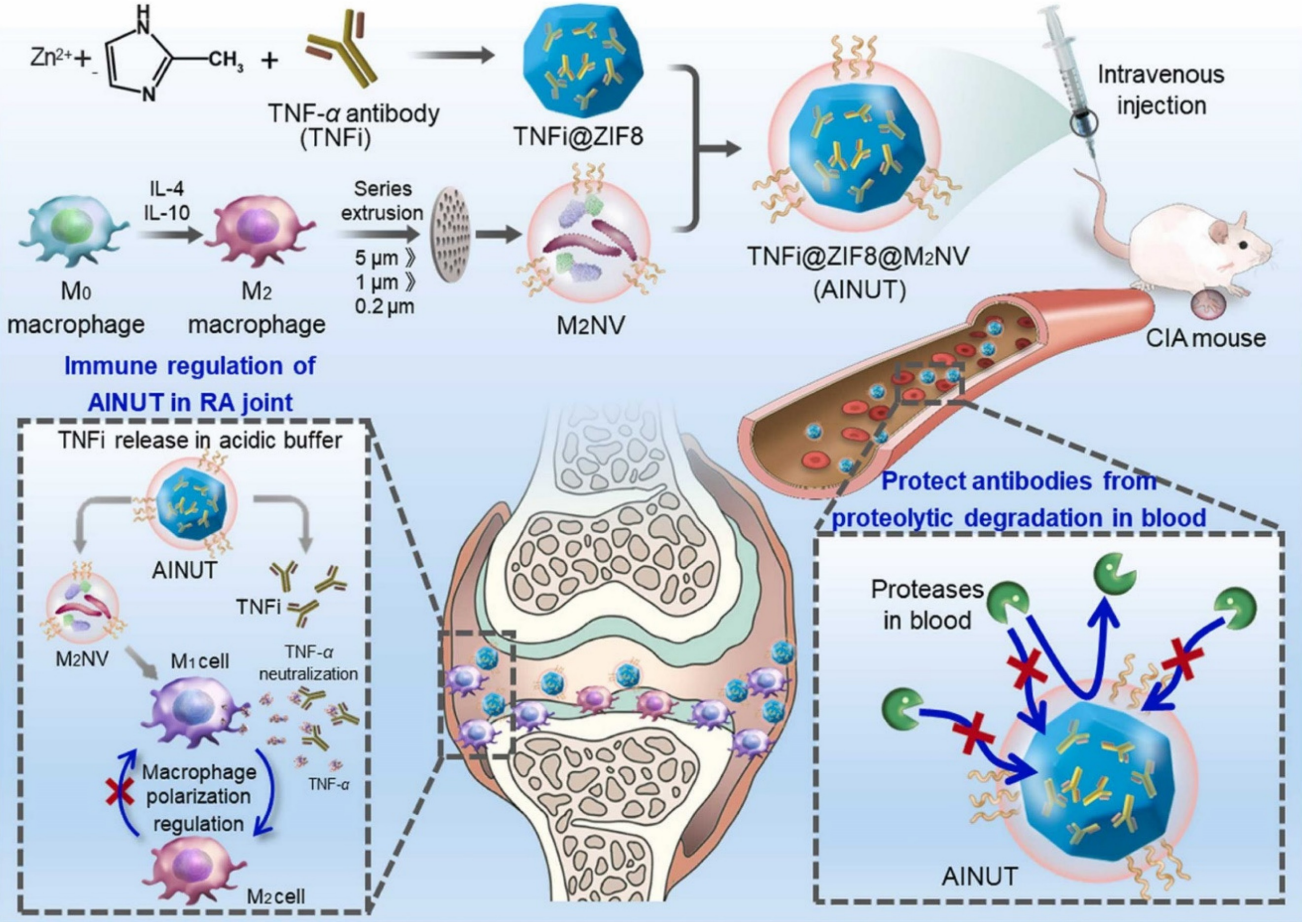
The fabrication and anti-inflammatory mechanism of AINUT. AINUT, a nanocomposite constructed with ZIF8 encapsulating the anti-TNF-α antibody TNFi, was enveloped in a nanocarrier derived from anti-inflammatory macrophages (M2NVs). The zeolitic imidazolate framework preserved the structural integrity and biological functionality of TNFi, even in the presence of proteases. In addition, the M2NV reduced the inflammatory response of the immune cells and attenuated toxic side effects. Adapted with permission, from 225 Copyright 2023 Elsevier.

Recently, cytokine-conjugated antibodies have shown promise in targeting and selectively accumulating at chronic inflammatory sites, achieving considerable progress in anti-inflammation therapy 225. However, the premature degradation of cytokine-conjugated antibodies remains a formidable challenge. To overcome this obstacle, Liu *et al.* devised an immunological nanonut, termed anti-TNF-α antibody-sheltered immunological nanonut (AINUT, ~200 nm, ~-20 mV) to mitigate RA progression 225 (Figure 9). AINUT, constructed with zeolitic imidazolate framework-8 (ZIF8) encapsulating the anti-TNF-α antibody (TNFi), was enveloped in a nanocarrier derived from anti-inflammatory macrophages (M_2_NVs). The nut-like structure preserved the structural integrity and biological functionality of TNFi, even in the presence of proteases. In addition, M_2_NVs modulated the immune microenvironment by redirecting inflammatory macrophages to anti-inflammatory pathways. Targeted delivery and dose reduction are the main methods to reduce the toxic side effects of AINUT. *In vivo*, AINUTs preferentially accumulated in acidic RA joints, enabling the sustained release of TNFi to neutralize TNF-α, thereby potently alleviating RA symptoms in a mouse model 225.

In addition to the protective cytokine, some antioxidant enzymes also play essential roles in inflammatory diseases 226, 227. The free radicals and other oxidants, which are critical yet damaging factors in the pathogenesis of inflammation, can be neutralized by these antioxidant enzymes 228. Notably, superoxide dismutase (SOD), as an antioxidant and anti-inflammatory enzyme, not only transforms superoxide into H_2_O_2_ and oxygen but also influences immune responses 228. To enrich SOD at inflammatory lesions without degrading protein drugs, Sun *et al.* synthesized several pH-sensitive copolymer-based nanomicelles to deliver SOD in a rat peritonitis model 227. Among them, nanomicelles composed of mPEG-poly (cyclohexane-1,4-dial acetone dimethyleneketal) (mPEG-PCADK) exhibited the most favorable properties, including high encapsulation efficiency, long blood circulation and accelerated acidolysis under acidic conditions. *In vivo*, mPEG-PCADK nanomicelles significantly suppressed leukocyte proliferation, providing a potent nano-carrier for the delivery of unstable protein drugs 227.

Peptide-based drugs have also garnered substantial attention due to several unparalleled advantages including quick synthesis and limited side effects 229, 230. Moreover, most therapeutic peptides can selectively target pathological lesions and regulate signaling pathways both *in vitro* and* in vivo*. However, the high polarity and small size of peptides generally lead to poor pharmacokinetics and availability 231. Formulating peptides into NPs offers a potential solution to these drawbacks. For instance, Keum *et al.* designed biomimetic disc-shaped lipid NPs encapsulated with cell-penetrating 9-arginine-modified peptides (APTstat3-9R@DLNP, ∼20-30 nm) 232. APTstat3-9R@DLNP demonstrated excellent colloidal stability over a period of two weeks, without any noticeable alteration in its hydrodynamic size. Additionally, the presence of biomimetic lipid components facilitated transmembrane permeation, resulting in enhanced penetration effects. In a mouse model of pulmonary fibrosis, APTstat3-9R@DLNPs effectively mitigated pulmonary inflammation via the inhibition of multiple inflammatory mediators and STAT3 activation. Such a therapeutic nanoparticle serves as a safe and efficient nanoplatform for delivering peptide drugs 232.

### Self-therapeutic nanotherapeutics

Self-therapeutic nanomaterials are a type of nanomaterials that modulate inflammation without external drugs, acting as anti-inflammatory agents or interacting with the biological environment. Inorganic NPs such as Au NPs 233, Pt NPs 234 and CeO_2_ NPs 235 and organic nanomaterials such as hydrogel possess intrinsic therapeutic properties against inflammation 236. Some nanomaterials show antioxidant-like property to mimic the functions of natural ntioxidant-like catalase (CAT), glutathione peroxidase (GPx) and SOD, which can hinder oxidation in inflamed tissues or cells by trapping free radicals or reducing oxidation initiation 237. Notably, the self-therapeutic nanomaterials simplifies design complexity, eliminating concerns about drug encapsulation and release 71. Consequently, they hold potential cost and translational advantages, requiring no extra chemical or biological components that might complicate large-scale production and purification 71.

AuNPs have emerged as fascinating candidates for self-therapeutic nanoplatforms with unique physicochemical properties, including cell targeting and cytokine expression regulation 233. AuNPs can inhibit pro-inflammatory cytokines and their tunable properties for precise adjustment of therapeutic outcomes 233. To study the impact of NPs' size and alkylation on efficacy, Han *et al.* proposed a series of alkyl-terminated Au NPs for the prevention and treatment of psoriasis 233. Upon comparing different Au NPs, they found that Au NPs (Au_3_@PEG-octadecyl_30%_) with 3 nm gold cores modified with polyethylene glycol with 30% octadecyl chains could effectively penetrate into the epidermis and interact with keratinocytes. In contrast, unalkylated PEG-coated Au cores are >20 nm, hindering skin penetration. Additionally, 3 nm PEG-coated cores with >30 mol% octadecyl chains mildly aggregate to form ~35 nm clusters, limiting effective skin penetration. Furthermore, Au_3_@PEG-octadecyl_30%_ worked by inhibiting key genes associated with the IL-17 signaling pathway, known for its connection to excessive skin cell growth and inflammation. Compared with commercial steroid and vitamin D analog treatments, Au_3_@PEG-octadecyl_30%_ exhibited comparable efficacy with fewer side effects such as hair loss and skin wrinkling, providing a safe and efficient therapy for psoriasis 233. Au NPs can also inhibit other inflammatory signaling pathways. For instance, Chan *et al.* introduced a sub-10-nm Au NP (~7 nm) coated with PEG and folic acid (FA) for kidney fibrosis 238. The outcomes demonstrated that the Au NP was more effective in reducing tissue degeneration and inhibiting the p38α enzyme compared to standard Captopril therapy 238. Shifting macrophage polarization from M1 to M2 plays key roles in resolving inflammation 239. Lu *et al.* invented glutathione-protected gold nanoclusters (GA) for treating IBD 239. Remarkably, GA exhibited targeted accumulation in the colon. GA not only facilitated M2 differentiation of IL-4-treated peritoneal macrophages but also shifted macrophage polarization from M1 to M2 in a pro-inflammatory setting 239.

Apart from common metal compounds, transition metal selenides such as ruthenium (Ru) has emerged as promising catalytic materials 237. Ru is a transition metal element with multiple oxidation states, and the interconversion between Ru^2+^/Ru^3+^ redox pairs can be used to eliminate ROS 240. For instance, Deng *et al.* introduced a cobalt selenide-based biocatalyst with an amorphous Ru@CoSe nanolayer (thickness: 2.5 nm, -25.8 mV) for rapid ROS elimination 237. The nanolayer, created by doping Ru atoms onto CoSe nanorods through a solvothermal method and subsequent high-energy ball milling, offered enriched electrons and unoccupied orbitals from Ru, enabling outstanding pseudo-peroxidase kinetics. These Ru species served as “regulators,” adjusting Co site electron states and oxygen interactions, enhancing reversible redox properties at catalytic sites, outperforming many existing metal compound-based ROS biocatalysts 237. In a diabetic mouse model with skin wounds, Ru@CoSe demonstrated superior ROS-scavenging capabilities in inflammatory diabetic wounds 237. Additionally, Ru complexes have shown efficacy in neutralizing reactive nitrogen species (RNS) 240. Wang *et al.* incorporated transition metal atoms (Ru and Ni) to create ultrathin trimetallic two-dimensional nanosheets (TMNSs, thickness: 2.4-4.8 nm, -21.5 mV) 241. The modification facilitated RNS and ROS elimination by forming Ru-O and Ni-O bonds on the TMNSs' surface, effectively addressing chronic colitis inflammation 241.

Some metal oxide NPs have also been used as self-therapeutic agents, including iron oxide (Fe_3_O_4_) 242, CeO_2_ 234 and zinc oxide 243. Among them, CeO_2_ is recognized for its antioxidative and ROS scavenging properties by switching between two oxidation states, Ce^3+^ and Ce^4+^ 71. CeO_2_ NPs can mimic the functions of natural antioxidases, such as CAT and SOD, allowing the decomposition of ROS and achieving anti-inflammatory efficacy 71. For instance, Min *et al.* developed a straightforward noble metal deposition strategy, depositing Au NPs, Ag NPs (5-10 nm) and Pt NPs on the surface of hollow mesoporous CeO_2_ nanospheres 234. These hybrid structures, combining the multienzyme-like activities of CeO_2_ and ROS scavenging capacities of metal NPs, demonstrate high efficacy in IBD and ear inflammatory mice models via the downregulation IL-1β and TNF-α 234. Among them, CeO_2_@Ag displayed exceptional therapeutic effectiveness at a dosage of 0.5 mg/kg 234.

Metal-organic frameworks (MOFs) integrated with nanozymes show outstanding antioxidant abilities, offering potential as anti-neuroinflammatory agents for treating inflammation. Jiang *et al.* deliberately chiral MOFs incorporated with nanozymes by incorporating ultra-small Pt nanozymes (Ptzymes, 6-8 nm) into L- and D-chiral imidazolate zeolite frameworks (Ptzyme@L-ZIF (82.2 nm, 25.1 mV) and Ptzyme@D-ZIF (70.4 nm, 18.4 mV)) through shell-ligand exchange reactions 244. Both Ptzyme@L-ZIF and Ptzyme@D-ZIF showed strong SOD- and CAT-like activities. Compared to Ptzyme@L-ZIF, Ptzyme@D-ZIF exhibited increased accumulation in the brain lesions of Parkinson's disease (PD) mouse model due to its prolonged plasma retention time and diverse pathways across the BBB 244. Furthermore, Ptzyme@D-ZIFs effectively inhibited neuroinflammation-induced apoptosis and ferroptosis, leading to superior therapeutic effects 244. Additionally, Liu *et al*. developed a Ru-based metal-organic framework (RuMOF, 141.6 nm) as a nano-antioxidant for treating inflammation-related diseases 240. Such a Ru-MOF system demonstrated excellent biocompatibility and potent catalytic activity, which eliminated excessive reactive oxygen and nitrogen species (RONS) and blocked TNF-related inflammatory pathways in both inflammation-related conditions (LPS-induced endotoxemia and dextran sulfate sodium (DDS)-induced colitis) 240.

Additionally, some organic nanomaterials also play important roles in fighting inflammation. For instance, hydrogel can hinder oxidation in inflamed tissues or cells by trapping free radicals or reducing oxidation initiation 236. Moreover, hydrogel can also provide mechanical support and promote tissue regeneration by mimicking the extracellular matrix (ECM) 236. Conley *et al.* developed a dynamic nanohybrid peptide hydrogel (NHPH) to modulate the inflammatory and inhibitory microenvironment of the intervertebral disc (IVD) and promote its repair and regeneration 236. The NHPH, formed through self-assembly of peptide amphiphiles with biodegradable 2D nanomaterials, exhibited desirable enzyme-like functions. Notably, NHPH could scavenge ROS, remodel the ECM and provide sustained delivery of growth and differentiation factors. In a rat nucleotomy model, the NHPH demonstrated improved nucleus pulposus cell differentiation and inflammation reduction, suggesting potential for treating IVD degeneration and fibrocartilaginous injuries 236.

Despite their potential, self-therapeutic nanomaterials encounter challenges including toxicity, immunogenicity and biodistribution 245. Addressing these issues requires further studies to comprehend the mechanisms of specific inflammation, optimize the synthesis and assess the safety tests for self-therapeutic nanomaterials.

### Summary

In summary, nano-DDSs have significant advantages in enhancing drug efficacy and reducing side effects. For chemical drugs, nano-DDSs improve their solubility, bioavailability and stability, while minimizing side effects via targeted delivery. Gene drugs demonstrate high targeting and specificity, enabling the regulation of gene expression for challenging-to-treat diseases, such as autoimmune diseases and viral infections. However, they face challenges, such as membrane crossing, easy degradation and high immunogenicity. Encapsulating gene drugs into NPs facilitates intracellular release, improving the biological activity and safety. Protein or peptide drugs, known for their high biocompatibility and biological activity, can simulate or regulate physiological functions, but with stability concerns. Nano-DDSs serve as biocompatible vehicles, precisely delivering various protein or peptide agents to inflammatory areas for effective treatments. There are also self-therapeutic nanomaterials that can act as anti-inflammatory or antioxidant agents independently to treat inflammation, eliminating concerns about drug encapsulation and release.

Nano-DDSs, while offering advantages, also face challenges including complex preparation, high cost, uneven biological distribution, unclear clearance mechanisms and insufficient toxicity evaluation. For example, liposomes have good application potential, but the lipid bilayer structure is not stable, leading to hydrolysis and leakage during the transport of chemical or gene ingredients 246. Biodegradable polymers such as PLGA are commonly used for RNA drug delivery, but the* in vivo* metabolism and toxicity after inhalation are often overlooked, which may lead to challenges for anti-inflammatory nanomedicine, especially in chronic conditions requiring repetitive administrations 247.

Despite the challenges, there has been continuous progress in developing nano-DDSs for anti-inflammatory treatments. To manage the rational design of novel nano-drugs, researchers need to explore various approaches, including: i) Identifying pathologic hallmarks or pathways in different inflammatory diseases, focusing on nanomaterials sensitive to biochemical signals like pH, ROS and enzymes at diseased sites. ii) Modifying the surface of NPs to enhance uptake by target cells and impart diverse biological functions. iii) Co-delivering multiple therapeutic agents with different targets to enhance biomedical performance. Additionally, from a translational perspective, achieving biodegradable, large-scale, high-throughput manufacturing processes for nano-DDSs is also crucial.

## Combination strategies

**Figure 10:**
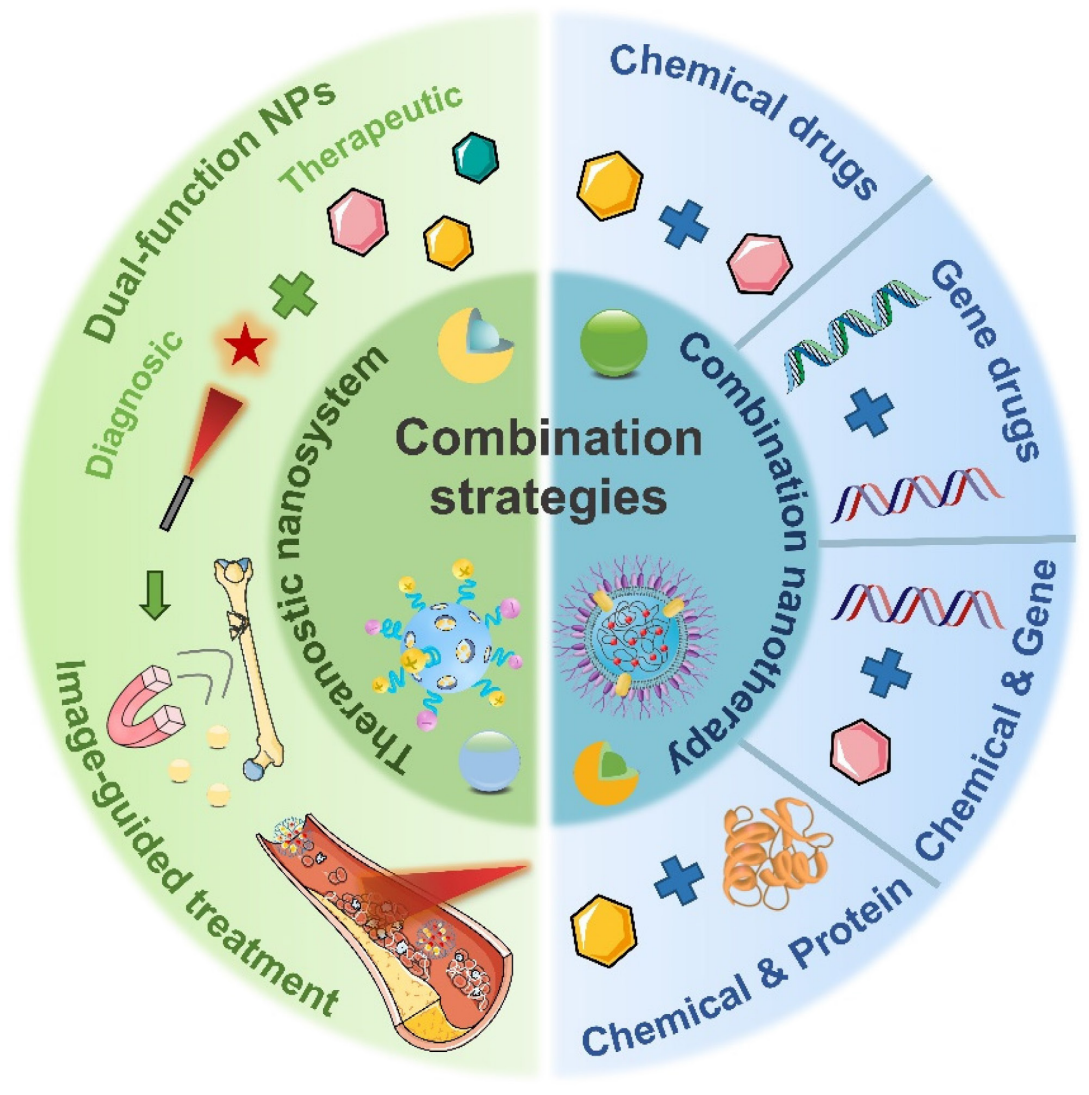
Schematic representation of multiple strategies for anti-inflammatory nanotheranostics and combination nanotherapeutics.

With advances in medical treatment and technology, an increasing number of scientists are devoted to developing diagnostic and therapeutic agents with reduced treatment frequencies 248. Theranostics, a term combining therapy and diagnostics, offering imaging-guided treatment and therapeutic effect monitoring, has gained significant attention. In addition, compared with single therapy modalities, combined therapies exhibit better therapeutic efficacy in inflammation therapy 249. Recently, nanomaterials with appropriate size, outstanding targeting capabilities and controllable release, have become optimal carriers for co-delivering two or more components 5, 250, 251. As depicted in Figure 10, in this section, we will introduce several representative nanocarriers for theranostics and combined therapy.

### Theranostic nanosystem

**Figure 11:**
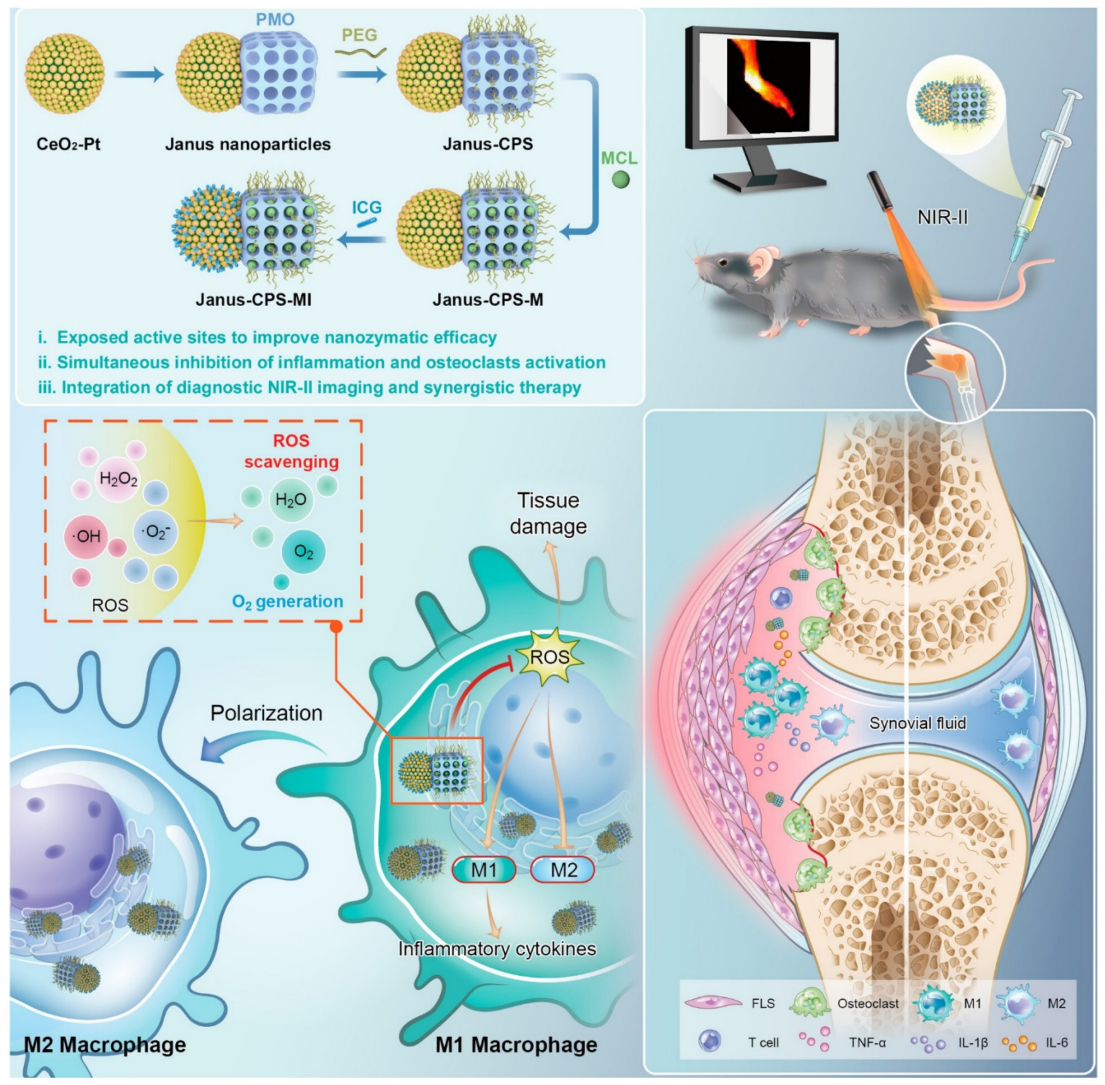
The structure and theranostic mechanism of Janus-CPS. Janus-CPS was a peanut-shaped Janus nanoplatform with CeO_2_-Pt nanozymes on one side and PMO on the other side. The CeO_2_-Pt nanozymes could scavenge ROS and the PMO could load an anti-inflammatory drug to regulate inflammation. *In vivo*, Janus-CPS loaded with ICG, a NIR-II fluorophore, could visualize the inflamed joints and blood vessels in a CIA mouse model. Moreover, Janus-CPS loaded with MCL, an anti-osteoclast drug, could reduce the bone erosion and inflammation in the CIA mice by inhibiting the formation and activity of osteoclasts. Adapted with permission, from 257 Copyright 2023 American Chemical Society.

Most inflammatory disorders are prevalent chronic diseases with clinical silence and delayed therapy 252. Thus, timely detection and treatment of inflammation are imperative. Theranostic NPs, which provide both diagnostic and therapeutic functions in a single dose, have emerged as significant contributors to the biomedical field 253. Recently, a series of nano-DDSs have been developed for inflammation theranostics 152, 254-256. For example, Liu* et al.* created a peanut-shaped Janus nanoplatform named Janus cationic polymer-silica (Janus-CPS, ~100 nm), for the early theranostics of RA 257 (Figure 11). This Janus nanoplatform consisted of a CeO_2_-Pt nanozyme subunit on one side and a high-capacity periodic mesoporous organosilica (PMO) on the other. Notably, the Janus nanostructure enhanced ROS scavenging with increased active sites, showing augmented capabilities compared to core-shell NPs. Moreover, Janus-CPS loaded with ICG demonstrated remarkable NIR-II imaging capabilities, enabling early detection of RA in a CIA mouse model. Additionally, micheliolide (MCL), a plant-derived compound with anti-osteoclastogenesis effects, was also loaded to synergize anti-inflammatory properties for effective treatment of RA via macrophage polarization, ROS scavenging and antiosteoclast properties 257.

In addition to FL, MRI examination is also deemed as a potent tool for monitoring therapeutic effects via the measurement of relaxation times 79. To visualize inflamed lesions, NPs have been extensively employed to enhance MRI signals 79. For instance, Liu *et al.* developed dual-functional arginine (Arg) and Mn^2+^-doped polydopamine NPs (DAMM NPs, 229.1 nm) to load Dex for diagnosing and treating OA 79. *In vitro*, DAMM NPs continuously released Dex effectively, inhibiting synovial inflammation and toll-like receptor 3 (TLR-3) production in chondrocytes and chondrocyte apoptosis through the TLR-3/NF-κB pathway. Additionally, DAMM NPs played a significant role in removing ROS generated in chondrocytes, thereby inhibiting ROS-induced M1 macrophage polarization and chondrocyte apoptosis. Notably, DAMM NPs, when chelated with Mn^2+^, exerted robust T1-T2 MRI contrast that enabled the potential tracking of nanoparticle retention at the injured cartilage. *In vivo*, DAMM NPs demonstrated enhanced MRI contrast capabilities and desirable performance in ameliorating OA development in the joint cavity of mice, enabling traceable delivery of nanomedicines 79.

### Combination nanotherapeutics

Notwithstanding significant advancements in inflammatory treatment, the effectiveness of monotherapy is limited. Combination nanotherapy for inflammation employs multifunctional nanosystems and a variety of therapeutic agents (such as chemical drugs, gene drugs and protein drugs) to amplify the effectiveness of anti-inflammatory interventions 258, 259.

The combination of two chemical drugs simultaneously via a nanocarrier has been demonstrated to enhance drug delivery, increase therapeutic effectiveness and mitigate dose escalation-induced toxicity 260. For instance, ticagrelor is an antiplatelet agent that inhibits platelet aggregation, while simvastatin is a statin that curtails the polarization of M1-type macrophages and down-regulate inflammatory-associated cytokines 259. To achieve the synergistic AS therapeutic effect, Zhao *et al.* developed a fibronectin-targeted nanoparticle to co-deliver simvastatin and ticagrelor 259. Firstly, a ROS-responsive nanoprodrug (TPTS, 185 nm, -4 mV) was synthesized by conjugating an α-tocopherol polyethylene glycol derivative and simvastatin with a thioketal linker. Subsequently, CREKA-containing lipopeptides, ticagrelor and TPTS were co-assembled into NPs (TPTS/C/T) via hydrogen bonding interactions. With the assistance of CREKA peptides, TPTS/C/T could specifically target atherosclerotic lesions and release both ticagrelor and simvastatin in conditions of excessive ROS, fostering a synergistic anti-inflammatory effect. Compared with TPTS and free statin groups, TPTS/C/T exhibited superior anti-inflammatory efficacy and a better safety profile in an apolipoprotein E-deficient (ApoE^-/-^) mouse model, providing a novel avenue for synergistic treatment of inflammation 259. Various chemical drugs-based combination strategies for anti-inflammatory nanotherapy are also explored in Table 4.

Aside from the suboptimal efficacy of mono-chemical drugs in treating inflammation, single-gene therapy, which primarily targets individual inflammatory pathways, is also insufficient for reversing the progression of inflammation 250, 265, 266. Inflammation is typically regulated by intricate signaling pathways, such as NF-κB, MAPK and JAK/STAT 265. Therefore, delivering different gene-drug combinations targeting separate pathways or multiple different inflammatory mediators effectively dampens the inflammatory cascade, leading to a more comprehensive and sustained therapeutic outcome. For example, the pathogenesis of immunoglobulin A nephropathy (IgAN) is associated with the pivotal involvement of the p38 MAPK and NF-κB signaling pathways. To achieve concurrent inhibition, Wang *et al.* reported a liposomal siRNA delivery system (2i@DuaLR, ~110 nm, 6.6 mV) to alleviate glomerulonephritis-related inflammation by simultaneously delivering p38α MAPK inhibitor and p65 siRNA in the glomerulus 267. With the assistance of PEI, p65 siRNA and p38α MAPK siRNA were simultaneously encapsulated into a liposomal nanoparticle and further modified with PEG and octa-arginine. Owing to the suitable size and special charge reversal, 2i@DuaLR could achieve superior glomerulus targeting and selective accumulation at the targeted sites. More importantly, a mere 4 nmol/kg 2i@DuaLR was found to be as efficient as a high dosage of traditional anti-inflammatories in an IgAN mouse model, while maintaining low systemic toxicity. Such a strategy offers an effective therapeutic nanoplatform for treating glomerulus-related inflammation 267.

**Figure 12:**
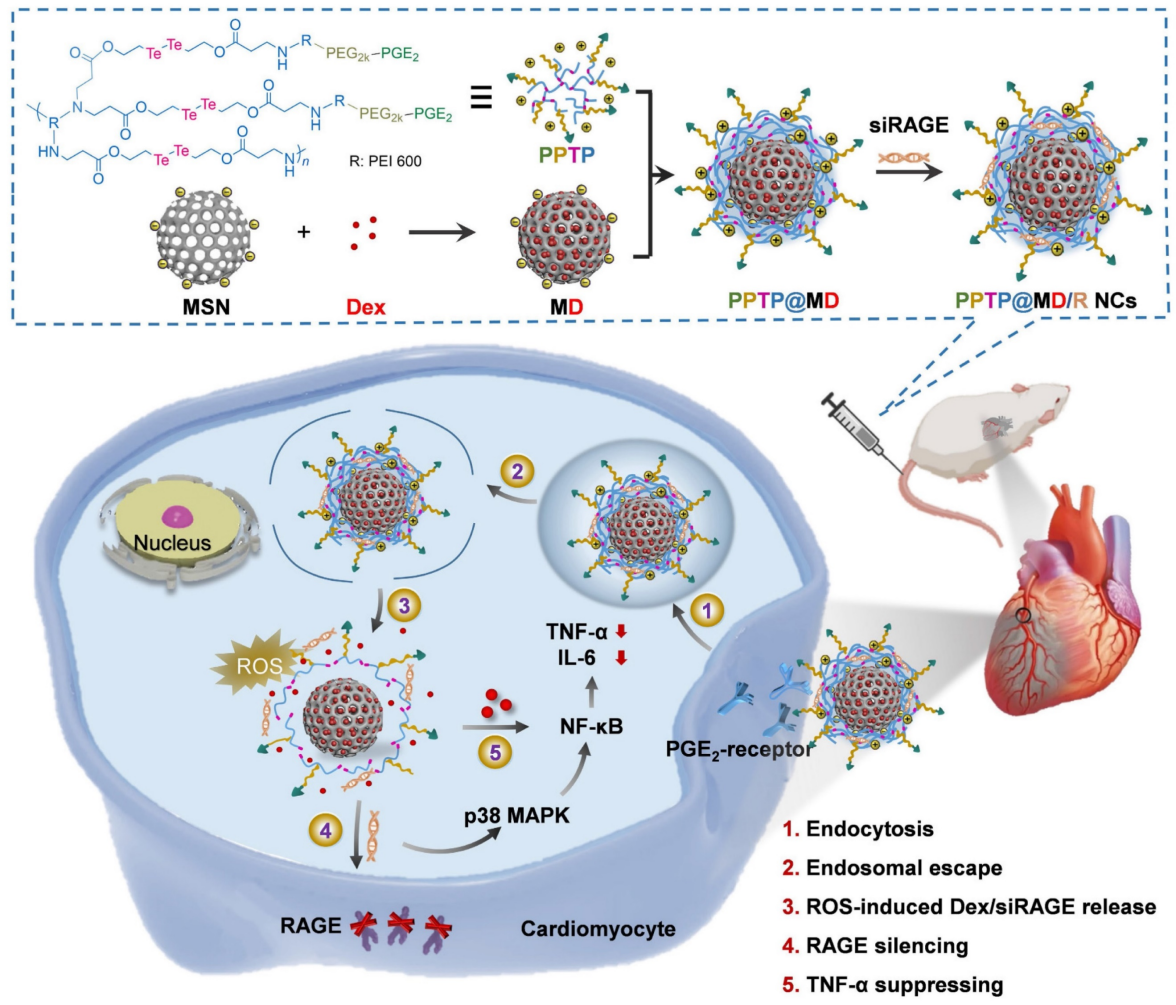
The ROS-ultrasensitive MSNs to concurrently deliver siRAGE and Dex for the synergistic treatment of myocardial inflammation. The MSNs were coated with PPTP, which further complexed with siRAGE and suppressed the pre-leakage of Dex. Upon injection into a myocardial IR-injured rat model, the nanomedicine selectively targeted the inflamed cardiomyocytes, reduced myocardial infarct size, improved cardiac function and attenuated inflammation via RAGE silencing (72%) and a synergistic anti-inflammatory therapeutic effect. Adapted with permission, from 5 Copyright 2022 Springer Nature.

In addition to the aforementioned combination therapies, therapeutic gene agents can also synergize with chemical drugs to exert a potent anti-inflammatory response 5. Among them, gene therapies are capable of modifying or silencing the expression of pro-inflammatory genes, while chemical drugs can inhibit the activity of inflammatory mediators. Concurrent use of gene-based and chemical drugs may have a synergistic effect, surpassing the sum of their contributions 5. As shown in Figure 12, Lan *et al.* strategically engineered ROS-ultrasensitive mesoporous silica NPs (MSNs, ~200 nm, -18 mV) to simultaneously deliver RAGE siRNA (siRAGE) and Dex for the synergistic treatment of myocardial inflammation 5. Upon the encapsulation of Dex within the MSN cavities, a PGE_2_-PEG-modified tellurium-crosslinked polyethyleneimine (PPTP) was employed to encapsulate the MSNs for preventing Dex pre-leakage and facilitating siRNA condensation.

Intracellularly, PPTP underwent degradation into smaller segments in response to excessive ROS, thereby facilitating the release of siRAGE and Dex. This mechanism led to the achievement of efficient receptor for advanced glycation end-products (RAGE) silencing (72%) and a synergistic anti-inflammatory therapeutic effect. Upon intravenous administration, the nanomedicine selectively targeted the inflamed cardiomyocytes, reduced myocardial infarct size, improved cardiac function and attenuated inflammation in a myocardial ischemia-reperfusion (IR)-injured rat model 5.

Compared with chemical and gene drugs, protein drugs exhibit superior target affinity and safety. Nevertheless, relying solely on protein therapy might be insufficient for complex inflammatory responses 268. The combination of protein drugs and chemical drugs could be a favorable strategy. For example, to sustain the mechanical function of the IVD and mitigate inflammatory reactions, Han *et al.* constructed a biomimetic core-shell nanofibrous scaffold to co-load ibuprofen (IBU) and transforming growth factor beta 3 (TGFβ3) by using electrospinning technology 269. Specifically, hyaluronic acid (HA) hydrosol was employed to encapsulate TGFβ3 and then was transformed into an emulsion to load IBU by dispersing in a spinning formulation. During the process of electrospinning, HA micro-droplets within the emulsion were further encapsulated into Poly (L-lactic acid) (PLLA) fibers to attain a dual-drug co-loaded core-shell nanofibrous scaffold. The dual-drug delivery system was able to assemble into an angle-ply structure, forming a highly biomimetic annulus fibrosus (AF). *In vivo*, due to the rapid release of IBU and sustained release of TGFβ3, the inflammatory lesions were improved and a nascent ECM was formed. Notably, the excellent anti-inflammatory capabilities and biocompatibility of this biomimetic nanofibrous scaffold were demonstrated in two rat tail defect models, suggesting potential applications for IVD repair 269.

### Summary

Based on the above-mentioned, various multifunctional nanoplatforms have been developed for inflammation thernostics and combination therapy. For theranostics, NPs play a crucial role in image-guided inflammation therapy, enabling real-time visualization of drug accumulation in inflamed sites and optimizing the dose of drug treatment. For combination therapy, co-encapsulating multiple bioactive anti-inflammatory agents in nano-DDSs enhances therapeutic efficacy, reduces drug resistance and dose-related toxicity, provides insights into pharmacokinetics and supports personalized medicine therapy. Given that each imaging and therapeutic modality has unique advantages and limitations, selecting an optimal combination and dose levels are crucial, as they can ensure enhanced therapeutic efficacy and reduced toxicity.

## Conclusions and perspectives

This review focuses on the recent and notable advances of various nanomaterials in the diagnosis and treatment of inflammation. So far, there have been lots of nano-DDSs developed for inflammation diagnosis and therapy. Notably, some of the NPs have been approved by the FDA. For example, ferumoxytol (Feraheme^®^) is a SPIO NP (30 nm), which was approved by the FDA in 2009 for the treatment of iron deficiency anemia 270. In addition, ferumoxytol is also explored as an off-label MRI contrast agent, particularly for the clear imaging of blood vessels 268. For the treatment of inflammation, fenofibrate (Tricor^®^) is a nanocrystal product approved in 2004 for preventing AS, which show a 9% increase in oral bioavailability compared to micronized Tricor 271. More recently, the rapid development of LNPs has facilitated the systematic siRNA and mRNA delivery, with the FDA approving for mRNA-1273 (Moderna) and BNT162b2 (Pfizer-BioNTech) to prevent COVID-19 272.

Indeed, the clinical application of nanomaterials still faces challenges. First, safety stands as a fundamental criterion for clinical nanoformulations. For instance, the widely used biodegradable polymer PLGA may trigger inflammation *in vivo* due to its degradation byproducts and interactions with macrophages. Second, animal models of chronic inflammation often fall short of clinical standards. Most *in vivo* studies testing inflammation diagnosis and therapy rely on early-stage animal models, with limited involvement of larger animals or clinical trials. Finally, scaling up nanomedicine production encounters hurdles in manufacturing and controls (CMC) as well as regulatory requirements, which influence clinical translation.

Despite the challenges in clinical translation, nanomaterials still hold great promise for advancing the inflammation diagnosis and treatment. Thus, the optimization of nanomaterials is urgently needed. For diagnosis, targeting biomedical materials is essential to enhance concentration in the target organ for better resolution. Additionally, ensuring rapidly metabolizing doses or degradable formulations to avoid excessive nanomaterials accumulation in the body is also crucial. In terms of treatment, designing biochemical stimuli-DDSs based on the inflammatory types and sites could be helpful to minimize side effects and improve efficacy. Furthermore, considering the varying levels of oxidative stress and the different signaling pathways in different inflammation-related diseases and the various stages of inflammation, targeting multiple signaling pathways could be effective. Notably, it is very necessary to build clinically applicable *in vivo* studies to identify therapeutic efficiency and address the potential risks. Finally, optimizing the regulatory frameworks as well as ensuring the consistent manufacturing and toxicology assessments are vital for expediting the clinical research and approvals. For example, the regular calibration of nanosensors and nanoprobes is the guarantee for inflammation diagnosis accuracy. Additionally, the accurate and consistent compositions, sizes, synthesis methods, dosages and administration routes of nano-DDSs are also key to ensure the inflammation therapeutic efficacy.

In conclusion, recognizing and addressing the challenges in nano-DDSs for the diagnosis and treatment of inflammation is crucial for efficient clinical translations. In the future, collaborative efforts from diverse fields will be beneficial to advance nanomaterials from the lab to clinical applications, ultimately improving the life quality and saving more lives.

## Figures and Tables

**Table 1:** Representative nanosensors for detection of inflammation in 2021-2023.

Platform	Analyte	Transducer	Linear range	Detection limit	Disease	Year	Refs.
Platinum nanoclusters	H_2_O_2_	Fluorescent and volumetric chip	1-500 μM	/	IBD	2022	84
Colloidal quantum dots	MPO	Amperometric	0.001-1 ng/ml	31.6 fg/ml	/	2023	85
Ag_2_S quantum dots deposited Bi_2_S_3_ nanorods,	PCT	Electrochemical	0.5-50 pg/ml	0.18 pg/ml	/	2023	86
AuNPs	Lipoproteinassociated phospholipase A_2_	Electrochemical	/	0.21 ng/ml	AS	2023	87
Ionic liquid crystal, carbon nanotubes and Fe-Ni alloy nanoparticles	H_2_O_2_	Electrochemical	0.007-1000 μM	0.971 nM	/	2021	88
Graphene quantum dots and AuNPs aggregate-embodied copolymer hydrogel	Cardiac troponin-I	Electrochemical	1-1000 pg/ml	0.1 pg/ml	Myocardial infarction	2021	89

**Table 2:** Representative nanoprobes for imaging of inflammation in 2021-2024.

Imaging modality	Nanoprobe	Inflammation	Route	Advantages	Disadvantages	Year	Refs.
**FI**	QMT-CBT	AD	I.V.	Enhanced fluorescence (AIE signal); Turn on and near-infrared imaging; Reduced autofluorescence interference	Limited stability and biocompatibility; Low clinical applicability	2023	[162
	Ir-CBM	Epilepsy	I.V.	Two-photon excitation; Ratiometric luminescence; Long-lived emission; High selectivity; Low cytotoxicity *in vivo*	Limited tissue penetration depth; Micellar environment restrictions	2023	163
	PCN-NP-HPZ	AS	I.V.	Simultaneous sensing and imaging of pH and phosphorylation; High-resolution images	Potential toxicity; Limited specificity	2023	164
PAI	1-PAIN	Liver inflammation	I.V.	Deep tissue penetration Real-time monitoring; High selectivity and low background; High biocompatibility	Relatively low PA signal; Endogenous •OH and H_2_S interference	2022	104
	PA nanoagent	RA	I.V.	Deep tissue penetration High sensitivity and selectivity; Monitoring the therapeutic process; Enhance the PA conversion efficiency	Potential toxicity; Interference from other factors; Potential toxicity	2024	165
	L-CRP	AS	I.V.	High selectivity; Deep tissue penetration	Limited biodegradability	2023	166
MRI	TMSN@PM	Inflammation	I.V.	High selectivity; Non-invasive imaging; Real-time monitoring	Potential toxicity; Limited resolution and contrast	2022	[167
CT	PIDA nanofibers	IBD	Oral	Good compliance; Reduced scan time (within 2 h); Theranostics	Invasiveness; Limited penetration depth; Low spatial resolution	2023	168
	Exitron nano 12000	Abdominal aortic aneurysm	I.V.	Quantification of inflammation; Improved targeting and specificity	Invasiveness; Limited resolution and contrast	2021	138
FI & PAI	QY-SN-H_2_O_2_	IBD	Oral	Good compliance; High-resolution; Deep-penetration; ROS-responsiveness; Non-invasiveness	Limited stability and biocompatibilityy; Potential toxicity	2022	169
FI & PAI	MPN@CeOx	UC	Oral	Good compliance; ROS-responsiveness; Intestinal inflammation accumulation; Deep-penetration;	Potential toxicity (metal components)	2023	170
CT & MRI	BM@EP	UC	Oral	Good compliance; Colon-targeted delivery and controlled release; Quantitative and dynamic imaging; Improved accuracy and sensitivity	Limited stability and biocompatibility; Complexity for the synthesis	2022	171

**Table 3:** Representative small-molecule chemical drug-based nanotherapeutics in 2021-2023.

Platform	NP sizes (nm)	Zeta Potential (mV)	Drug	Route	Animal models	Mechanism	Year	Refs.
Polymeric NPs	TEM: 91.4	-17.4	Cinnamaldehyde	I.V.	CIA mouse model and Colitis mouse model	Scavenge ROS; Suppress NF-κB signal pathway	2023	199
	TEM: 171.7	-22.3	Magnolol	Oral	DSS-induced colitis mouse model	Scavenge ROS; Suppress NF-κB and signal pathway; Modulate gut health	2023	72
	DLS: 117.0	-18.6	Ginsenoside Rh2	Oral	DSS-induced colitis mouse model	Scavenge ROS; Suppress STAT3/miR-214 signal pathway	2022	73
Micelles	DLS: 190	-50.0	Rapamycin	I.V.	Atherosclerotic mouse mode	Scavenge ROS; Suppress pro-inflammatory factors	2023	199
	DLS: 88.1	-21.3	Curcumin	Oral	DSS-induced colitis mouse model	Suppress pro-inflammatory factors	2021	201
Nanogels	DLS: 124.2	-19.2	Phenytoin	I.V.	Status epilepsy rat model	Scavenge ROS	2023	202
	/	/	Losmapimod	Topical	Diabetic wound mouse model	Scavenge ROS; Enhance M2-type macrophage polarization; Suppress pro-inflammatory factors	2022	203
	/	/	Psoralen	Intra-articular injection	CIA mouse model	Improve bone homeostasis; Regulate metabolism; Suppress pro-inflammatory factors	2023	204
Liposomes	DLS: 85.6	-6.0	Celastrol	I.V.	Imiquimod-induced psoriasis mouse model	Inhibit maturation of DCs; Suppress pro-inflammatory factors	2022	205
Biomimetic NPs	DLS: 175.0	-20.0	Dexamethasone	I.V.	Endotoxin-induced lung inflammation murine model	Suppress pro-inflammatory factors	2021	206
	TEM: ~100	/	Indomethacin	Topical	CIA murine model	Suppress pro-inflammatory factors; Inhibit cyclooxygenase-2	2023	207

**Table 4:** Representative chemical drugs-based combination strategies for anti-inflammatory nanotherapeutics.

Platform	Drug 1	Drug 2	Route	Animal models	Mechanism	Year	Refs.
**Cationic** polypeptide nanomicelles	Losmapimod	Tempo	Topical	Dry eye mouse model-	Scavenge ROS; Suppress MAPK signal pathway	2022	261
ROS-responsive prodrug nanomicelles	Dexamethasone	Artesunate	I.V.	Adjuvant-induced arthritis rat model	Scavenge ROS; Suppress HIF-1α/NF-κB signal pathway; Enhance M2-type macrophage polarization	2022	262
mPEG-b-PCL nanomicelles	9-aminoacridine	Caffeic acid	I.V.	CIA rat model	Suppress HIF-1α/NF-κB signal pathway	2022	187
Self-assembled peptide hydrogel	3,5-dihydroxybenzoic acid	Ibuprofen	I.V.	Endotoxin-induced uveitis rabbit model	Scavenge ROS; Suppress pro-inflammatory factors (e.g., IL-1β, IL-6 and TNF-α); Suppress NF-κB and JAK-STAT signal pathways	2022	263
Injectable polymeric aggregate-embodied copolymer hydrogel	Dexamethasone	Tempo	Intra-articular injection	RA mouse model	Scavenge RNS an ROS; Upregulate immune cells	2021	264
